# Mitochondrial superoxide dismutase controls metabolic plasticity in pancreatic cancer

**DOI:** 10.1186/s12964-025-02555-8

**Published:** 2025-12-06

**Authors:** Sankaranarayanan Ramasubramanian, Rupert Öllinger, Carola Eberhagen, Hans Zischka, Roland M. Schmid, Henrik Einwächter

**Affiliations:** 1 Department of Medicine 2, School of Medicine and Health, Technical University of Munich, Ismaninger Straße 22, Munich, 81675 Germany; 2 Institute of Molecular Oncology and Functional Genomics, School of Medicine and Health, Technical University of Munich, Munich, Germany; 3 Institute of Molecular Toxicology and Pharmacology, Helmholtz Center Munich, German Research Center for Environmental Health, Ingolstädter Landstraße 1, Neuherberg, 85764 Germany; 4 Institute of Toxicology and Environmental Hygiene, School of Medicine and Health, Technical University Munich, Biedersteinerstraße 29, Munich, 80802 Germany

**Keywords:** Superoxide Dismutase (SOD2), Pancreatic Neoplasms, Myc Proto-Oncogene Proteins

## Abstract

**Background:**

The role of reactive oxygen species (ROS) in cancer is debated. One main antioxidant enzyme is mitochondrial superoxide dismutase (SOD2) which has been shown to influence tumor initiation and metastatic progression in several cancer types.

**Methods:**

To investigate the impact of *Sod2* deletion on pancreatic cancer biology and metabolism, we used CRISPR/Cas9 gene editing to generate 3 independent *Sod2-*deficient cell lines from murine Kras^G12D^ pancreatic cancer cell lines and analyzed them for proliferation, colony forming, mitochondrial respiration and RNA expression. In addition, mass spectrometry and isotope tracing were performed.

**Results:**

Proliferation and wound healing capacity were significantly impaired in *Sod2* deficient cell lines. Myc levels were significantly elevated in *Sod2*-deficient cells, and mitochondrial respiration was consecutively increased. This resulted in increased tolerance to glucose deprivation. Mechanistically, we detected a significantly reduced activity of succinate dehydrogenase (SDH) in *Sod2*-deficient cells. This resulted in increased peroxynitrite formation which was the cause of increased Myc activation.

**Conclusions:**

These findings reveal that *Sod2* shapes cellular metabolism in pancreatic cancer through peroxynitrite formation and Myc activation.

**Supplementary Information:**

The online version contains supplementary material available at 10.1186/s12964-025-02555-8.

## Background

Pancreatic ductal adenocarcinoma (PDAC) is one of the most aggressive cancers and remains largely incurable at the time of diagnosis. In the United States, pancreatic cancer now has the third-highest age-adjusted cancer mortality rate [1]. Patients with pancreatic cancer exhibit elevated markers of oxidative stress and decreased levels of reduced glutathione [2]. Studies investigating the role of reactive oxygen species (ROS) in pancreatic cancer suggest a complex relationship. Some reports indicate that ROS can induce apoptosis [3, 4] or cell cycle arrest [5] in pancreatic cancer cells. However, most studies support a pro-tumorigenic role for ROS in pancreatic cancer: Increased H_2_O_2_ levels have been detected in acinar-to-ductal metaplasia (ADM) induced by various stimuli, and the addition of catalase consistently blocked ADM [6]. Moreover, ROS have been found to exert anti-apoptotic effects in pancreatic cancer cells [7]. In line with this, mutant KRAS has been shown to induce ROS, with elevated ROS levels observed in PanIN and ADM lesions [8].

## Methods

### Culture and treatment of murine pancreatic cancer cell lines

Pancreatic cancer cell lines were derived from tumor tissues of mice harboring Kras^G12D^ mutations (Kras^G12D^ cell lines). These cells were maintained at 37 °C, 5% CO_2_ in a humidified incubator. Cell lines were cultured in pre-warmed standard glucose culture medium (DMEM, 41,965–062, Gibco) supplemented with 10% fetal calf serum (FCS), 1 × penicillin/streptomycin, and 1% MEM non-essential amino acids (glucose medium or basal culture). Once the cells reached confluence, they were passaged according to their respective proliferation rates. For biological assays, cancer cell lines were seeded into appropriate culture plates, and inhibitors were added to glucose medium at the required concentrations. The cells were then incubated for the designated time points. This study was conducted using established mouse cell lines and did not involve live animals or human subjects. Therefore, no ethical approval was required.

### Gene editing

The Cas9 double-nickase system was used for the generation of *Sod2*-CRISPR knock-out cells [9]. Guide RNAs (gRNAs) were designed using an online design tool (http://crispr.mit.edu/). Two pairs of 20-base-pair gRNAs sequences were selected to target exon 3 of the mouse *Sod2* gene (ENSMUSG00000006818) with a 48-base-pair offset. The gRNA sequences used as follows: *Sod2*-guideA (reverse): 5′-AGCCCGCGGCACCGGCCACA-3′ and *Sod2*-guideB (forward): 5′-GCCTTACGACTATGGCGCGC-3′. The gRNA oligonucleotides were annealed and cloned into pSpCas9n(BB)−2A-Puro (pX462, Addgene plasmid # 62,987, RRID:Addgene_62987) following the recommended protocol [10]. The pSpCas9n(BB)−2A-Puro-Sod2-guideA and -guideB constructs were co-transfected into three *Kras*^G12D^ cancer cell lines using the jetPRIME® transfection reagent (Polyplus-transfection SA, Illkirch-Graffenstaden, France). Following 48–72 h of puromycin selection, three individual clones were derived for each of the three control *Kras*^G12D^ cancer cell lines. Gene editing was confirmed using RT-PCR, immunoblotting and functional analysis via the SOD activity assay.

### Cell proliferation assay

Cancer cell lines were seeded in black 96-well plates (3603, Corning) at a density of 2,000 cells per well and allowed to attach overnight. To assess proliferation rates over 72 h, cells were plated in multiple wells to enable measurements at different time points. Once the cells had attached, appropriate treatments or culture conditions were applied to all plates, marking the 0 h (0 h) time point. At each designated time point (24 h, 48 h, and 72 h), the corresponding plates had their medium removed and were stored at −80 °C for a period ranging from a few hours to three days. Subsequently, the plates were thawed, and DNA content was measured using the CyQuant cell proliferation assay kit (C7026, Invitrogen) according to the manufacturer’s instructions. Fluorescence readings at 24 h, 48 h, and 72 h were normalized to 0 h fluorescence values to determine the proliferation rates of the cancer cell lines.

### Intracellular ATP measurement

Intracellular ATP levels were measured using the CellTiter-Glo assay kit (G7571, Promega). Cancer cell lines were seeded into black 96-well plates (3603, Corning) at a density of 10,000 cells per well and allowed to attach overnight. The normalized ATP levels to cell number, the same cancer cell lines were plated in duplicate in black 96-well plates (3603, Corning) for quantification using the CyQuant cell proliferation assay kit (C7026, Invitrogen) as previously described. After attachment, cancer cells were cultured under basal conditions and after 24 h of 60 µM 10058-F4 (F3680, Sigma-Aldrich) treatment, Glucose deprivation (4 mM glutamine), and Glutamine deprivation (25 mM Glucose). Following treatment, CTG reagent (G7571, Promega) was added to each well at a 1:1 ratio with the culture medium. The mixture was incubated for 10 min at room temperature protected from light, and luminescence was subsequently measured. ATP levels were normalized to cell number and expressed relative to control (*Kras*^G12D^) cancer cell lines.

### Measurement of oxygen consumption rate (OCR)

Mitochondrial respiration was assessed by measuring the oxygen consumption rate (OCR) using the XF96 Extracellular Flux analyzer (Seahorse Bioscience) as previously described [11]. Cancer cell lines were seeded at 7,000 cells per well in an XF96 plate (Seahorse Bioscience) and incubated for 24 h in a humidified 37 °C incubator with 5% CO_2_ in glucose medium. To normalize OCR values, duplicate wells of the same cancer cell lines were plated in 96-well plates (353,072, Falcon) for protein concentration estimation using Pierce™ BCA Protein Assay Kit (23,227, Thermo Fisher Scientific). Before OCR measurement, the culture medium was replaced with XF Assay medium (pH 7.4, Seahorse Bioscience) supplemented with 25 mM glucose, 1 mM sodium pyruvate, and 3.97 mM glutamine. Cells were then incubated in a CO_2_ –free incubator for 1 h at 37 °C. OCR values were normalized in protein concentration in each well. Various respiratory parameters were calculated as follows: Firstly, baseline OCR was measured and basal respiration was calculated as the difference between baseline OCR and non-mitochondrial respiration. ATP-linked respiration was determined by adding oligomycin (2.5 nM) and calculate the difference between baseline OCR and oligomycin-inhibited OCR. Maximum respiration was determined by adding CCCP (1 µM) along with pyruvate to drive the electron transport chain (ETC) to its maximal capacity. Maximum respiration was calculated by subtracting non-mitochondrial respiration from CCCP-stimulated OCR. Non-mitochondrial respiration was measured by adding antimycin A (2.5 µM) and rotenone (2.5 µM), inhibitors of complex III and complex I to completely inhibit mitochondrial respiration revealing non-mitochondrial OCR. Respiratory reserve capacity is a measure of the cell’s ability to adapt to stress or increased energy demands and this is calculated as the difference between maximum respiration and basal respiration.

### SOD activity assay

Mitochondrial enrichment from murine pancreatic cancer cell lines was performed using a modified version of an established protocol for cultured fibroblasts [12]. Cancer cell lines were seeded in 150 mm culture dishes and maintained at 37 °C, 5% CO_2_ until they reached 80–90% confluency, ensuring a cell yield of at least 5 × 10^6^ to 10^7^ cells to obtain a sufficient quantity of enriched mitochondria. Once 80–90% confluency was reached, cells underwent the mitochondrial enrichment procedure as described previously [12]. To ensure mitochondrial functionality remained intact, enriched mitochondria were used immediately for the SOD activity assay. The protein concentration of the mitochondria enriched fraction was determined using Pierce BCA Protein Assay Kit (23,227, Thermo Fisher Scientific). SOD activity was measured as mentioned in the manufacturer’s instruction (19,160, Sigma-Aldrich). SOD activity was then normalized to the protein concentration and activity expressed relative to control (*Kras*^G12D^) cancer cell lines.

### In vitro wound healing

Cancer cell lines were seeded in 6-well plates (353,224, Falcon) at a density of 3 × 10^5^ cells per well and cultured for 48 h until reaching 90% confluence. A wound was then introduced by scratching the monolayer with a P200 pipette tip, followed by medium replacement to remove cellular debris from the wound site. The initial wound area (0 h time point) was immediately imaged using 5 × magnification and wound closure was monitored by capturing images at the 24-h time point. Wound area was quantified using Fiji (RRID:SCR_002285) software, wound closure was analyzed over time.

### Western blot analysis

Protein lysates were fractionated by SDS-PAGE and transferred onto nitrocellulose membranes and probed with the following primary antibodies: MnSOD (ADI-SOD-111-F, 1:1000, Enzo Life Sciences, RRID:AB_10631750), HSP90 (#4874, 1:1000, Cell Signaling, RRID:AB_2121214), AMPKa (#2532, 1:1000, Cell Signaling), p-AMPKa (#2535, 1:1000, Cell Signaling), c-Myc (ab32072, 1:1000, abcam, RRID:AB_731658), and OXPHOS complexes I to V (ab110413, 1:250, abcam, RRID:AB_2629281). Proteins were detected using HRP-conjugated secondary antibodies, as per the manufacturer’s instructions: donkey anti-rabbit (NA9340V, 1:5000, GE Healthcare) or HRP-conjugated sheep anti-mouse (NA931V, 1:5000, GE Healthcare). Blots were developed using Amersham ECL Western Blotting Detection Reagent (RPN2106, GE Healthcare) and visualized with a Gel documentation system (CHEMIDOC XRS +, Bio-Rad, RRID:SCR_019690).

### Mass spectrometry and isotope tracing

Cancer cell lines were treated with 10 mM malonate (M1296, Sigma-Aldrich) and 60 µM 10058-F4 (F3680, Sigma-Aldrich) or maintained under basal conditions for 24 h. Following 24-h treatments, monolayer cells were washed with PBS and medium was replaced with either complete medium or glucose-free medium (Thermo Fisher Scientific) with 25 mM [^13^C_6_]-glucose (Sigma-Aldrich). Cells were incubated for 30 min, washed with cold PBS, harvested using a rubber cell scraper and frozen at −80 °C. Water-soluble metabolites were extracted with 0.5 ml ice-cold MeOH/H_2_O (80/20, v/v) containing 0.1 μM lamivudine (Sigma-Aldrich) and 0.1 μM sucrose (Sigma-Aldrich). After centrifugation, supernatants were transferred to an RP18 SPE column (Phenomenex) that had been activated with 0.5 ml CH_3_CN and conditioned with 0.5 mL of MeOH/H_2_O (80/20, v/v). The eluate was dried in a centrifugal evaporator (Savant) and dissolved in 50 μL 5 mM NH_4_OAc in CH_3_CN/H_2_O (50/50, v/v). Metabolites were analyzed by LC–MS using the following settings: For LC–MS analysis 3 μl of each sample was applied to a ZIC-cHILIC column (SeQuant ZIC-cHILIC, 3 μm, 100*2.1 mm). Metabolites were separated at 30 °C by LC using a DIONEX Ultimate 3000 UPLC system (Thermo Scientific) and the following solvents: Solvent A consisting of 5 mM NH_4_OAc in CH_3_CN/H_2_O (5/95, v/v) and solvent B consisting of 5 mM NH_4_OAc in CH_3_CN/H_2_O (95/5, v/v). At a flow rate of 200 µl/min, a linear gradient starting at 100% solvent B decreasing to 40% solvent B over 23 min was applied followed by 17 min constant elution with 40% solvent B, followed by a linear increase to 100% solvent B over 1 min. Recalibration of the column was achieved by 7 min prerun with 100% solvent B. All MS-analyses were performed on a high-resolution QExactive mass spectrometer (Thermo Scientific) in alternating positive- and negative full MS mode applying the following scan and HESI source parameters: Scan Range: 69.0—1000 m/z. Resolution: 70,000, AGC-Target: 3E6, Maximum Injection Time: 200 ms. Sheath gas: 30, auxiliary gas: 10, sweep gas: 3, Aux Gas Heater temperature: 120 °C. Spray voltage: 2.5 kV in positive ion mode and 3.6 kV in negative ion mode, Capillary temperature: 320 °C, S-lens RF level: 55.0. Signal determination and quantitation was performed using TraceFinder™ Software Version 3.3 (Thermo Fisher). All analyses were performed with three independent biological replicates.

### RNASeq analysis

Library preparation for bulk-sequencing of poly(A)-RNA was performed as described previously [13]. Briefly, barcoded cDNA of each sample was generated with a Maxima RT polymerase (Thermo Fisher) using oligo-dT primer containing barcodes, unique molecular identifiers (UMIs) and an adaptor. Ends of the cDNAs were extended by a template switch oligo (TSO) and full-length cDNA was amplified with primers binding to the TSO-site and the adaptor. NEB UltraII FS kit was used to fragment cDNA. After end repair and A-tailing a TruSeq adapter was ligated and 3’-end-fragments were finally amplified using primers with Illumina P5 and P7 overhangs. In contrast to [13], the P5 and P7 sites were modified to allow sequencing of the cDNA in read1 and barcodes and UMIs in read2 to achieve a better cluster recognition. The library was sequenced on a NextSeq 500 (Illumina) with 67 cycles for the cDNA in read1 and 16 cycles for the barcodes and UMIs in read2. Data processing was carried out using the published Drop-seq pipeline (v1.0) to generate sample- and gene-wise UMI tables [14]. The GRCm38 reference genome was used for alignment, and transcript and gene definitions were used based on GENCODE version M25.

### Gene Set Variation Analysis (GSVA)

RNASeq data were normalized with the DEseq2 package in R. Gene Set Variation analysis (GSVA) was performed using the gsva package in R with the MSigDB Hallmark gene set collection.

### Myc transcriptional activity

Cancer cells were treated with 60 µM 10058-F4 (F3680, Sigma-Aldrich), 10 mM Malonate (M1296, Sigma-Aldrich), 100 µM MnTBAP (sc-221954A, Santa Cruz), and 1 mM L-NAME (S2877, Selleckchem) for 24 h. Following treatment, nuclear extracts from the treated and untreated (basal) culture cells were used to quantify Myc activation according to the manufacturer’s instructions provided with the c-Myc transcription factor assay kit (Abcam, ab207200). Myc transcriptional activity was then expressed as relative Myc activation comparing the treated cells to control (*Kras*^G12D^) cancer cell lines.

### Complex II activity

Cancer cell lines were seeded at a density of approximately 10^6^ cells in 150 mm culture dishes and maintained at 37 °C, 5% CO_2_ until the following day for attachment. Once the cells attached, they were cultured in standard glucose medium (basal culture) and treated with 100 µM MnTBAP (sc-221954A, Santa Cruz), 50 µM MitoTEMPO (HY-112879, Medchemexpress), 1 µM MitoQ (HY-100116A, Medchemexpress), 10 µM SKQ1 (HY-100474, Medchemexpress), and 10 mM Malonate (M1296, Sigma-Aldrich) for 24 h. After treatments, cells were detached using 0.05% trypsin–EDTA (25,300,054, Gibco). Trypsin activity was inhibited with medium, and the cell suspension was centrifuged (1000xg for 5 min at 4 °C). The resulting cell pellet was washed with DPBS (14,190,169, Gibco) followed by another centrifugation step (1000xg for 5 min at 4 °C). The resulting cell pellet was washed, followed by another centrifugation step at the same conditions to collect the cells. The cell pellet was resuspended in 20 mM hypotonic potassium phosphate buffer (pH 7.5) and the suspension was subjected to several cycles of suction and expulsion using a Hamilton syringe until a homogenous solution was obtained. The resulting cell lysate was snap frozen in liquid nitrogen and thawed at 37 °C three times. The lysates were kept on ice for subsequent complex II activity analysis and an aliquot of 15 µl was used for protein concentration determination with the Pierce™ BCA Protein Assay Kit (23,227, Thermo Fisher Scientific). Complex II activity was determined according to a previously published protocol [15] and expressed as enzyme activity (nmol x min^−1^ × mg^−1^) using the following formula:

$$\begin{aligned} \mathrm{Activity}\;&(\mathrm{nmol}\;\mathrm x\;\min\nolimits^{-1}\;\mathrm x\;\mathrm{mg}^{-1})\;=\;\\&(\mathrm\Delta\;\mathrm{Absorbance}/\min\;\times\;1,000)\\&/\;\lbrack(\mathrm{extinction}\;\mathrm{coefficient}\\&\;\times\;\mathrm{volume}\;\mathrm{of}\;\mathrm{sample}\;\mathrm{used}\;\mathrm{in}\;\mathrm{ml})\;\\&\times\;(\mathrm{sample}\;\mathrm{protein}\;\mathrm{concentration}\;\mathrm{in}\;\mathrm{mg}\;\mathrm x\;\mathrm{ml}^{-1})\rbrack \end{aligned}$$

### Electron microscopy

Cells were fixed with 2.5% glutaraldehyde (Science Services GmbH) and postfixed with 1% osmium tetroxide. After fixation, cells were dehydrated using acetone and embedded in epoxy resin. Ultrathin Sects. 60 nm were cut using the Reichert-Jung Ultracut E microtome. These sections were then stained with Uranyless (Science Services) and lead citrate. Images were acquired using a Jeol 1200 EXII electron microscope (Akishima, Tokyo, Japan) equipped with a KeenViewII digital camera (Olympus, Hamburg, Germany) and processed with the iTEM software package (analySIS Five; Olympus).

### Reactive oxygen species (ROS) measurement

Intracellular ROS levels were measured using CellROX Green reagent according to the manufacturer’s instructions (Invitrogen, C10444). Cancer cell lines were seeded in black 96-well plates (3603, Corning) at a density of 10,000 cells/well and allowed to attach overnight. For normalization, the same cancer cell lines were plated in duplicate on separate 96-well plates (3603, Corning) and cell number was measured using the CyQuant cell proliferation assay kit (C7026, Invitrogen) as previously described. After cell attachment, cells were treated with 100 µM MnTBAP and 10 mM Malonate for 24 h. Following treatment, the medium was removed, and cells were incubated with 5 µM CellROX Green in DMEM (with 10% FCS) for 30 min at 37 °C. After incubation, cells were washed with PBS, and fluorescence was measured using a plate reader with excitation at 485 nm and emission at 520 nm. The fluorescence values were normalized to cell number, and ROS levels were expressed relative to control (*Kras*^G12D^) cancer cell lines.

### Peroxynitrite measurement

Intracellular peroxynitrite levels were measured using the Abcam peroxynitrite assay kit (ab233468) according to the manufacturer's instructions. Cancer cell lines were seeded in black 96-well plates (3603, Corning) at a density of 50,000 cells/well and allowed to attach overnight. For normalization, the same cancer cell lines were plated in duplicate on separate 96-well plates (3603, Corning), and cell number was measured using CyQuant cell proliferation assay kit (C7026, Invitrogen) as described earlier. After cell attachment, the medium was replaced with fresh medium to initiate treatments as indicated (10 mM malonate, 100 µM MnTBAP, 0–500 µM L-NAME) for 2 h. The fresh medium contained both the culture medium (90 µL) and 10 µL assay solution (Peroxynitrite Sensor Green). Assay solution was prepared from a concentrated stock solution of Peroxynitrite Sensor Green. To make a concentrated stock solution of Peroxynitrite Sensor Green, 20 µL of DMSO was added to the vial containing the powder, resulting in a 500X stock concentration. The assay solution was made by adding 10 µL of the 500X stock with 500 µL of assay buffer provided with the kit. This mixture was then combined with the culture medium, and the cells were incubated at 37 °C, 5% CO_2_ in a humidified incubator for 2 h. Following the incubation period, fluorescence was measured using a fluorescence plate reader, with excitation at 490 nm and emission at 530 nm. The values were then normalized to cell number, and peroxynitrite levels were expressed relative to control (*Kras*^G12D^) cancer cell lines.

### 2-NBDG Glucose uptake assay

Cellular glucose uptake was measured using the Abcam 2-NBDG Glucose Uptake Assay Kit (ab235976) according to the manufacturer’s instructions. Cancer cell lines were seeded in black 96-well plates (3603, Corning) at a density of 50,000 cells/well and allowed to attach overnight. For normalization, the same cancer cell lines were plated in duplicate on separate 96-well plates (3603, Corning), and cell number was measured using CyQuant cell proliferation assay kit (C7026, Invitrogen) as described earlier. After cell attachment, the medium was replaced with fresh glucose-free medium. Ten minutes prior to 24 h of culture with glucose-free medium, 2-NBDG was added at a concentration of 100 µg/mL to each well and the cells were incubated with 2-NBDG for 10 min at 37 °C, 5% CO_2_ in a humidified incubator. After incubation, cells were washed twice with Cell-based assay buffer provided with the kit (ab235976). Following the washing step, fluorescence was measured using a fluorescence plate reader, with excitation at 485 nm and emission at 535 nm. The values were then normalized to cell number, and glucose uptake was expressed relative to control (*Kras*^G12D^) cancer cell lines.

### Mitochondrial membrane potential assay

Mitochondrial membrane potential was assessed using the TMRE Mitochondrial membrane potential assay kit (Cay701310-500, Cayman Chemical) following the manufacturer’s instructions. Briefly, cancer cell lines were seeded in black 96-well plates (3603, Corning) at a density of 50,000 cells/well and allowed to attach overnight. For normalization, the same cancer cell lines were plated in duplicate on separate 96-well plates (3603, Corning), and cell number was measured using CyQuant cell proliferation assay kit (C7026, Invitrogen) as described earlier. After cell attachment, the medium was replaced with fresh medium to initiate treatments (24 h of basal culture, glucose-free medium (4 mM glutamine), and glutamine-free medium (25 mM glucose), in combination with 60 µM 10058-F4, as indicated) for 24 h. 30 min prior to the end of the treatments, TMRE was added to the cells at a working concentration of 120 nM and the cells were incubated in the dark for 30 min at 37 °C, 5% CO_2_ in a humidified incubator. Following this incubation period, cells were washed twice with Assay buffer provided with the kit (Cay701310-500, Cayman Chemical). Following the washing step, plates were equilibrated to room temperature for 15 min and fluorescence was measured using a fluorescence plate reader, with excitation at 530 nm and emission at 580 nm. The values were then normalized to cell number, and mitochondrial membrane potential was expressed relative to control (*Kras*^G12D^) cancer cell lines.

### Data collection

Normalized counts and associated clinical information for PACA-AU [16] were obtained from the International Cancer Genome Consortium (ICGC) Data Portal. Data for TCGA-PAAD [17] were retrieved from the Broad Institute's GDAC Firehose platform. The results presented here are in part based on data generated by the TCGA Research Network: https://www.cancer.gov/tcga.

### Statistical analysis

No randomization or group allocation was applicable, as all experimental conditions were predefined. The graphical arrangement of data and statistically testing for significance was performed using GraphPad Prism 8 (GraphPad Software, RRID:SCR_002798). For parametric data, statistical comparisons were made using Student's t-tests.

### Supplementary material and methods

#### Reverse Transcription (RT) – PCR

Total RNA of cancer cell lines was isolated using Maxwell® 16 LEV simplyRNA Tissue Kit (AS1280, Promega) and Maxwell® 16 Instrument (Promega) as per manufacturer’s instructions. 1 µg of RNA was reverse transcribed with SuperScript II. PCR was performed with the following primers:

**Table:** 

Target gene	Primer Sequence
Cyclophilin A	5’-ATGGTCAACCCCACCGTG-3’ 5’-TTCTGCTGTCTTTGGAACTTTGTC-3’
*Sod2*	5’-ACACATTAACGCGCAGATCA-3’ 5’-ATATGTCCCCCACCATTGAA-3’

### Pyruvate Dehydrogenase/PDH Activity assay

Pyruvate dehydrogenase activity was assayed using the colorimetric Pyruvate Dehydrogenase Activity Assay Kit (NBP3-25,783, Bio-Techne) according to the manufacturer’s instructions. Briefly, cancer cell lines were seeded at a density of approximately 10^6^ cells in 150 mm culture dishes and maintained at 37 °C, 5% CO_2_ until the following day for attachment. Following attachment, medium was replaced and the cells were allowed to grow for 24 h. Following the 24-h incubation period, cells were harvested, washed with PBS, and lysed in cold extraction buffer. Lysates were centrifuged at 10,000 × g for 10 min at 4 °C, and the supernatants were collected for PDH activity analysis and protein concentration determination with the Pierce BCA Protein Assay Kit (23,227, Thermo Fisher Scientific). Absorbance was measured using a plate reader at 450 nm after 20 s and 200 s from a 96-well plate by incubating cell lysates with the assay reagents provided with the kit (NBP3-25,783, Bio-techne). PDH activity was calculated from the change in absorbance (ΔA) using the standard curve and the values were normalized to total protein concentration.

### GSH/GSSG assay

The GSH/GSSG ratio was determined using the measurement of reduced glutathione (GSH) and oxidized glutathione (GSSG) following the manufacturer’s instructions using the GSH/GSSG-Glo™ Assay kit (V6612, Promega).

## Results

### Low *Sod2* expression correlates with improved prognosis in pancreatic cancer patients

Given the complex and often debated role of ROS and antioxidant systems in pancreatic cancer, we analyzed two publicly available datasets, PACA-AU and TCGA PAAD. Our goal was to determine whether the expression of genes associated with the Gene Ontology (GO) term GO:0016209 ("antioxidant activity") could serve as a significant predictor of overall survival in pancreatic cancer patients. Across both datasets, we identified 76 genes with antioxidant functions (see table S1). Of these, only six genes exhibited a statistically significant and consistent correlation with patient survival. Among them, *Sod2* stood out as the only enzyme localized to the mitochondria and its high expression was linked to poor predicted survival outcomes (Fig. S1A and S1B). Based on these observations, we aimed to further investigate the impact of *Sod2* deletion in established cancer cell lines.

### ***Sod***2-deficient ***Kras***^G12D^ cell lines show increased ROS and reduced proliferation

**Fig. 1:**
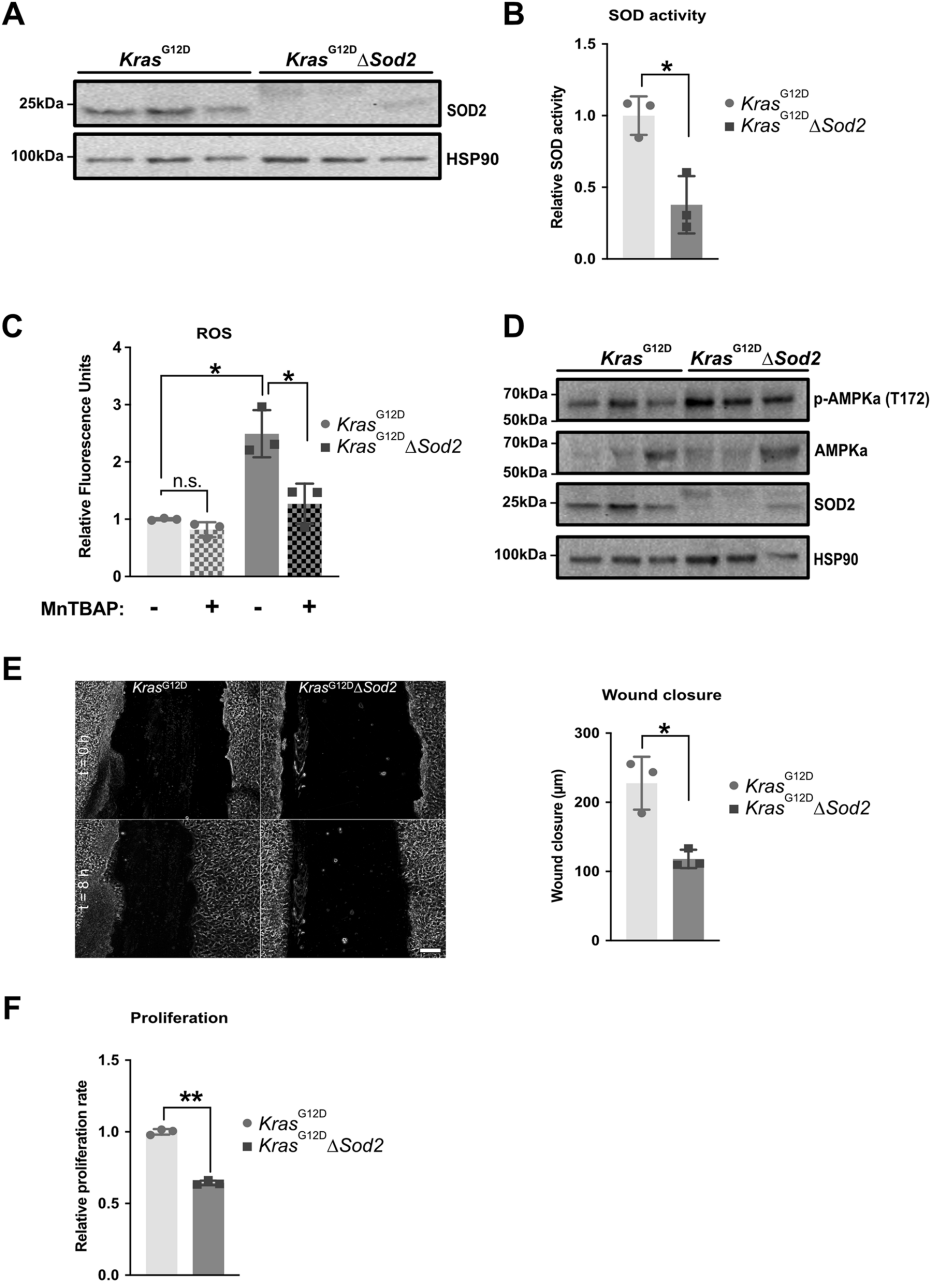
Sod2-deficient Kras^G12D^ cell lines show increased ROS and proliferate less. **A** Western blot for SOD2 of 3 Kras^G12D^ control cell lines and 3 Kras^G12D^∆*Sod2* cell lines, **B** SOD activity in 3 Kras^G12D^ control cell lines and 3 Kras^G12D^∆*Sod2* cell lines **C** ROS levels of 3 Kras^G12D^ control cell lines and 3 Kras^G12D^∆*Sod2* cell lines after 24-h culture in standard glucose medium (basal condition) and 100 µM MnTBAP addition. **D** Western blot for AMPK and pAMPK in 3 Kras^G12D^∆*Sod2* cell lines compared to 3 Kras^G12D^ control cell lines. **E** Representative images of wound healing assay at t = 0 h and t = 8 h from 3 Kras^G12D^ control cell lines and 3 Kras^G12D^∆*Sod2* cell lines and quantitative analysis of wound healing assay, scale bar, 200 µm. **F** Relative proliferation rate of 3 Kras^G12D^ control cell lines and 3 Kras.^G12D^∆*Sod2* cell lines after 72-h culture in standard glucose medium (basal culture). Error bars are SD, all *p*-values were calculated using Student’s t-test for paired samples. *, *p* < 0.05, **, *p* < 0.01

To investigate the effects of *Sod2* loss, we selected three independent *Kras*^G12D^ mutant pancreatic cancer cell lines previously isolated from genetically engineered KC mice [18]. Using a CRISPR/Cas9 approach, we successfully targeted the *Sod2* locus, with untransfected cell lines serving as controls. Sequencing confirmed successful editing, resulting in a truncated *Sod2* sequence in two clones and the insertion of a 102 bp repeat sequence in one clone (Fig. S1C). Reduced SOD2 expression was confirmed at both the protein level (Fig. 1A) and at the RNA level via RT-PCR (Fig. S1D). Additionally, mitochondrial SOD activity was significantly lower in the edited clones (Fig. 1B). As a result, ROS levels were significantly elevated in *Sod2*-deficient cells but could be reduced by treatment with the SOD-mimetic MnTBAP (Fig. 1C). The GSH/GSSG ratio was lower in *Sod2*-deficient cells compared to controls, though this difference did not reach significance (Fig. S1E). Further analysis revealed increased phosphorylation of AMPK, a key metabolic and redox sensor, in *Sod2*-deficient cells (Fig. 1D).

To assess the phenotypic consequences of *Sod2* deletion, we examined cell migration and proliferation. Wound healing capacity was significantly impaired, and proliferation rates were reduced by 35% in *Sod2*-deficient cells (Fig. 1E/F). Despite these functional changes, overall morphology remained unaltered (Fig. S1F-K).

### Increased Myc activity in ***Kras***^G12D^∆***Sod2*** cell lines mediates tolerance to glucose deprivation

**Fig. 2:**
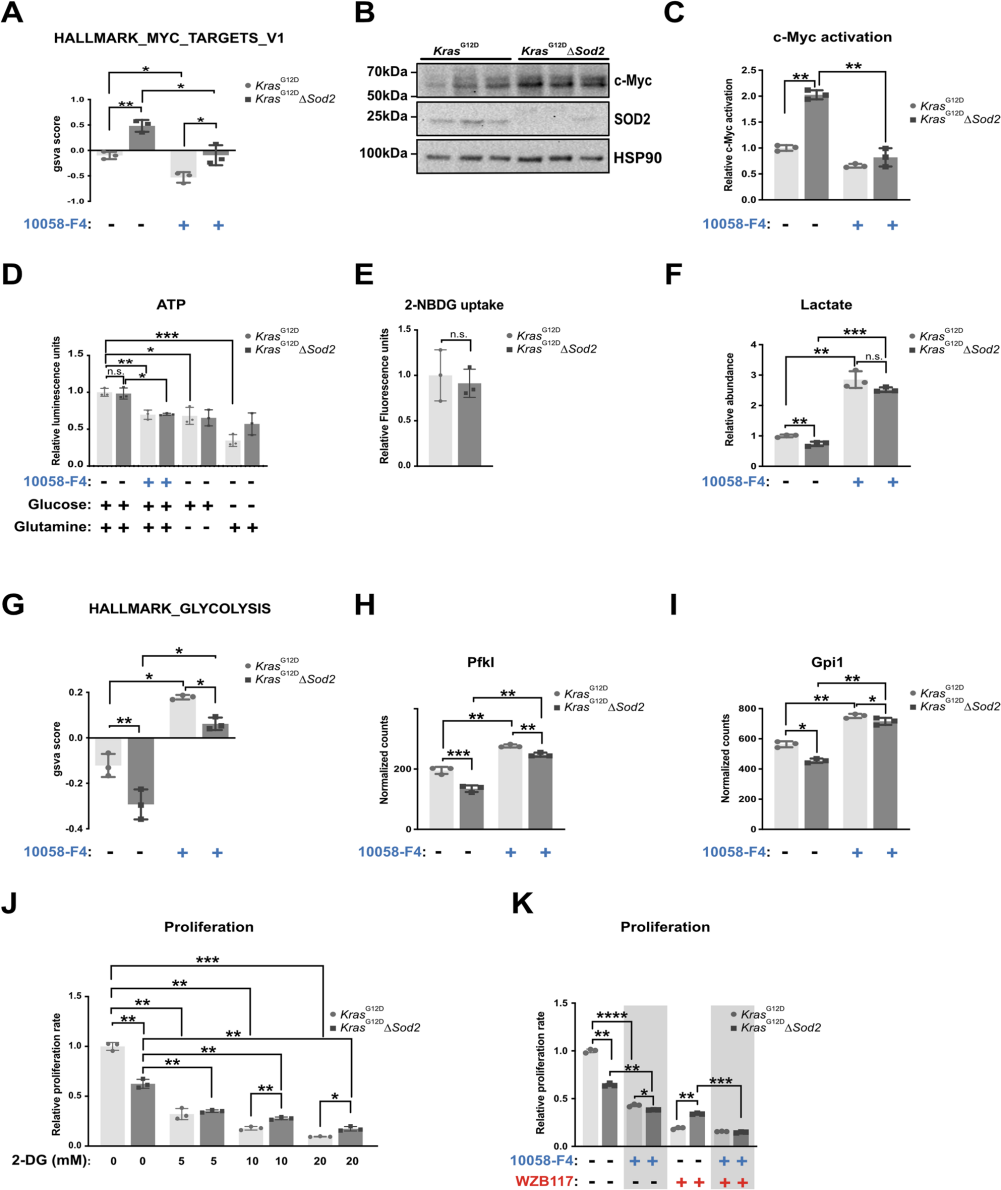
Increased Myc in *Kras*^G12D^∆*Sod2* cell lines mediates tolerance to glucose deprivation. **A** Enrichment scores for the geneset HALLMARK_MYC_TARGETS_V1 in RNA-Seq data from 3 *Kras*^G12D^ and 3 *Kras*^G12D^*∆Sod2* cells with and without 60 µM 10058-F4 (Myc inhibition) treatment. **B** Western blot analysis for Myc in 3 *Kras*^G12D^ cell lines and 3 *Kras*^G12D^∆*Sod2* cells. **C** Myc transcriptional activation in 3 *Kras*^G12D^ and 3 *Kras*^G12D^∆*Sod2* cells after 24 h of basal culture and 60 µM 10058-F4 (Myc inhibition) treatment. **D** ATP levels in 3 *Kras*^G12D^ and 3 *Kras*^G12D^*∆Sod2* cells after 24 h of 60 µM 10058-F4 (Myc inhibition) treatment, Glucose-deprivation (4 mM glutamine), and Glutamine-deprivation (25 mM Glucose). **E** Glucose uptake measured using 2-NBDG uptake assay from 3 *Kras*^G12D^ and 3 *Kras*^G12D^*∆Sod2* cells** F** Lactate abundance after 24 h of basal culture and Myc inhibition with 60 µM 10058-F4. **G** Enrichment scores for the geneset HALLMARK_GLYCOLYSIS in RNA-Seq data from 3 *Kras*^G12D^ and 3 *Kras*^G12D^*∆Sod2* cells with and without 60 µM 10058-F4 (Myc inhibition) treatment. **H**,** I** Normalized gene expression counts for glycolysis-associated genes PfkI (**H**) and Gpi1 **(I)** in 3 *Kras*^G12D^ and 3 *Kras*^G12D^∆*Sod2* cells in the presence and absence of 60 µM 10058_F4 (Myc inhibition)**. J** Relative proliferation rates of 3 *Kras*^G12D^ and 3 *Kras*^G12D^∆*Sod2* cancer cell lines after 72 h of basal culture and increasing doses of 2-deoxyglucose (2-DG), as indicated.** K** Relative proliferation rates of 3 *Kras*^G12D^ and 3 *Kras*.^G12D^∆*Sod2* cancer cell lines after 72 h of basal culture and with 30 µM WZB117 (Glut1 inhibition) and/or 60 µM 10058-F4, as indicated. Error bars are SD, p-value was calculated using Student’s t-test for paired samples. *, *p* < 0.05, **, *p* < 0.01, ***, *p* < 0.001, ****, *p* < 0.0001

To further explore the effects of *Sod2* deletion, we performed RNA-Seq analysis on both control and knockout cells, with and without treatment using the Myc inhibitor 10058_F4. Consistent with the increased Myc expression, the most significantly upregulated geneset among all HALLMARK genesets (see table S2) in *Sod2*-deficient cells was “HALLMARK_MYC_TARGETS_V1”. While Myc inhibition reduced the geneset score in both groups, the difference between *Sod2*-deficient cells and controls remained significant (Fig. 2A). We next examined the protein levels of Myc, a key regulator of ROS metabolism, and found a significant upregulation in *Sod2*-deficient cells (Fig. 2B). Elevated Myc protein levels and target gene expression corresponded with significantly increased Myc activation compared to control cells, and Myc inhibition for 24 h abolished Myc activity in both groups (Fig. 2C), confirming effective suppression of Myc transcriptional activity.

As Myc is known to regulate mitochondrial biogenesis [19] and bioenergetics [20]**,** we next assessed ATP levels under defined nutrient conditions. Under standard culture conditions, ATP levels did not differ between control and *Sod2*-deficient cells. Following Myc inhibition, ATP levels decreased in both groups without significant differences between genotypes. Removal of glutamine from glucose-containing medium did not further reduce ATP levels compared to Myc inhibition alone. In contrast, glucose withdrawal in the presence of glutamine caused an additional ATP reduction in control cells, whereas ATP levels in *Sod2*-deficient cells remained unchanged. To exclude altered glucose uptake as an underlying cause, we performed 2-NBDG uptake measurements. Glucose uptake was not significantly different between control and *Sod2*-deficient cells (Fig. 2E). Unexpectedly, lactate levels were significantly lower in *Sod2*-deficient cells and Myc inhibition significantly increased lactate production in both groups (Fig. 2F). This contrasts with the established role of transcriptional activation of key glycolytic enzymes by Myc [21]. Interestingly, the "HALLMARK_GLYCOLYSIS" geneset (Fig. 2G) mirrored the expression pattern of "HALLMARK_MYC_TARGETS_V1" (Fig. 2A). To further investigate glycolytic regulation, we analyzed the expression of genes associated with the GO term GO:0006096_("glycolysis"). The most significantly regulated genes between *Sod2*-deficient cells and controls were *Pfkl* and *Gpi1*. Myc inhibition led to a significant increase in the expression of both genes in control and *Sod2-*deficient cells (Fig. 2H and Fig. 2I).

These findings suggest that Myc plays a broader role in pancreatic cancer cell metabolism beyond its established function in glycolysis activation. To further assess glycolytic dependency, we quantified proliferation of control and *Sod2*-deficient cells under basal conditions and upon treatment with increasing concentrations of 2-DG. While 2-DG led to a dose-dependent reduction in proliferation in both groups, *Sod2*-deficient cells were significantly less impaired at the two highest concentrations (Fig. 2J), indicating reduced sensitivity to glycolytic inhibition. To test whether this phenotype extends to inhibition of glucose uptake, we next treated the cells with WZB117, a Glut1 inhibitor known to block glucose uptake and inhibit glycolysis [22]. In line with their lower lactate levels and their decreased sensitivity to 2-DG, *Sod2*-deficient cells exhibited increased tolerance to Glut1 inhibition, which was completely reversed when Myc inhibition was combined with WZB117 (Fig. 2K). These findings suggest that Myc mediates increased tolerance to glucose deprivation in *Sod2*-deficient cells, potentially by altering mitochondrial functionality rather than simply enhancing glycolysis.

### Myc mediates increased mitochondrial respiration in ***Sod***2-deficient ***Kras***^G12D^ cell lines

**Fig. 3:**
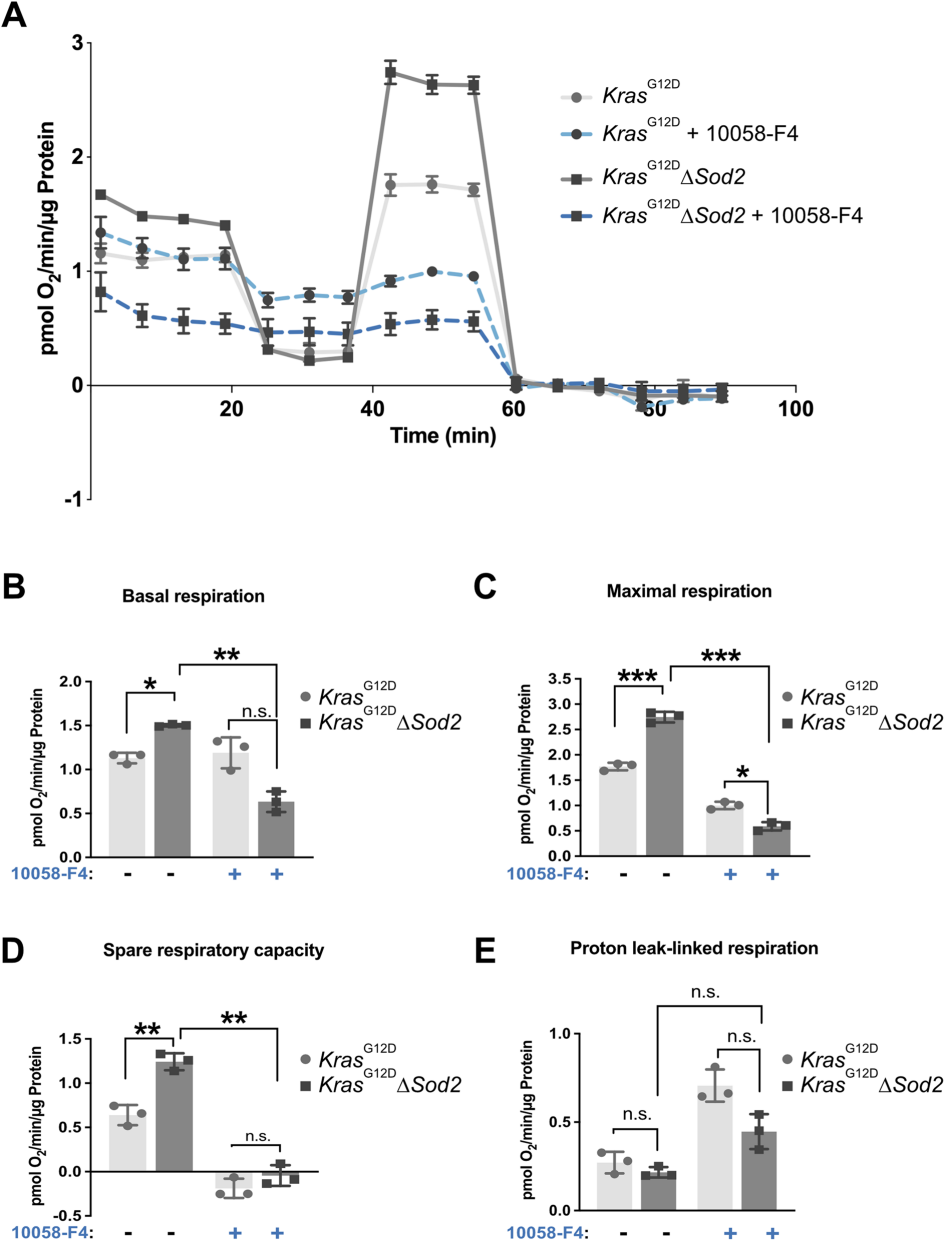
Myc regulates increased mitochondrial respiration in *Sod*2-deficient *Kras*^G12D^ cell lines. **A** OCR analysis with and without 24 h of Myc inhibition with 60 µM 10058-F4 in *Kras*^G12D^ cell lines and *Kras*.^G12D^∆*Sod2* cell lines. Individual respiratory rate parameters calculated from OCR showing the effect of 24 h of Myc inhibition on mitochondrial respiration: **B** basal, **C** maximal respiration, **D** reserve/spare respiratory capacity, and **E** proton leak. Error bars are SD, *p*-value was calculated using Student’s t-test for paired samples. *, *p* < 0.05, **, *p* < 0.01, ***, *p* < 0.001

To assess mitochondrial respiration, we measured Oxygen Consumption Rate (OCR) [23]in *Sod2*-deficient and control cells under basal culture conditions, as well as following treatment with Myc inhibitor (Fig. 3A). Analysis of the OCR data revealed that *Sod2*-deficient cells exhibited significantly increased basal OCR (Fig. 3B) maximum OCR (Fig. 3C), and spare respiratory capacity (Fig. 3D) compared to control cells. Notably, these enhancements in mitochondrial respiration were completely abolished upon Myc inhibition, indicating a Myc-dependent effect. Interestingly, proton leak linked respiration (Fig. 3E) showed no significantly differences between *Sod2*-deficient and control cells, suggesting that this parameter is not influenced by Myc activity.

### Complex II (SDH) inactivation mimics the effects of *Sod*2-deficiency

**Fig. 4:**
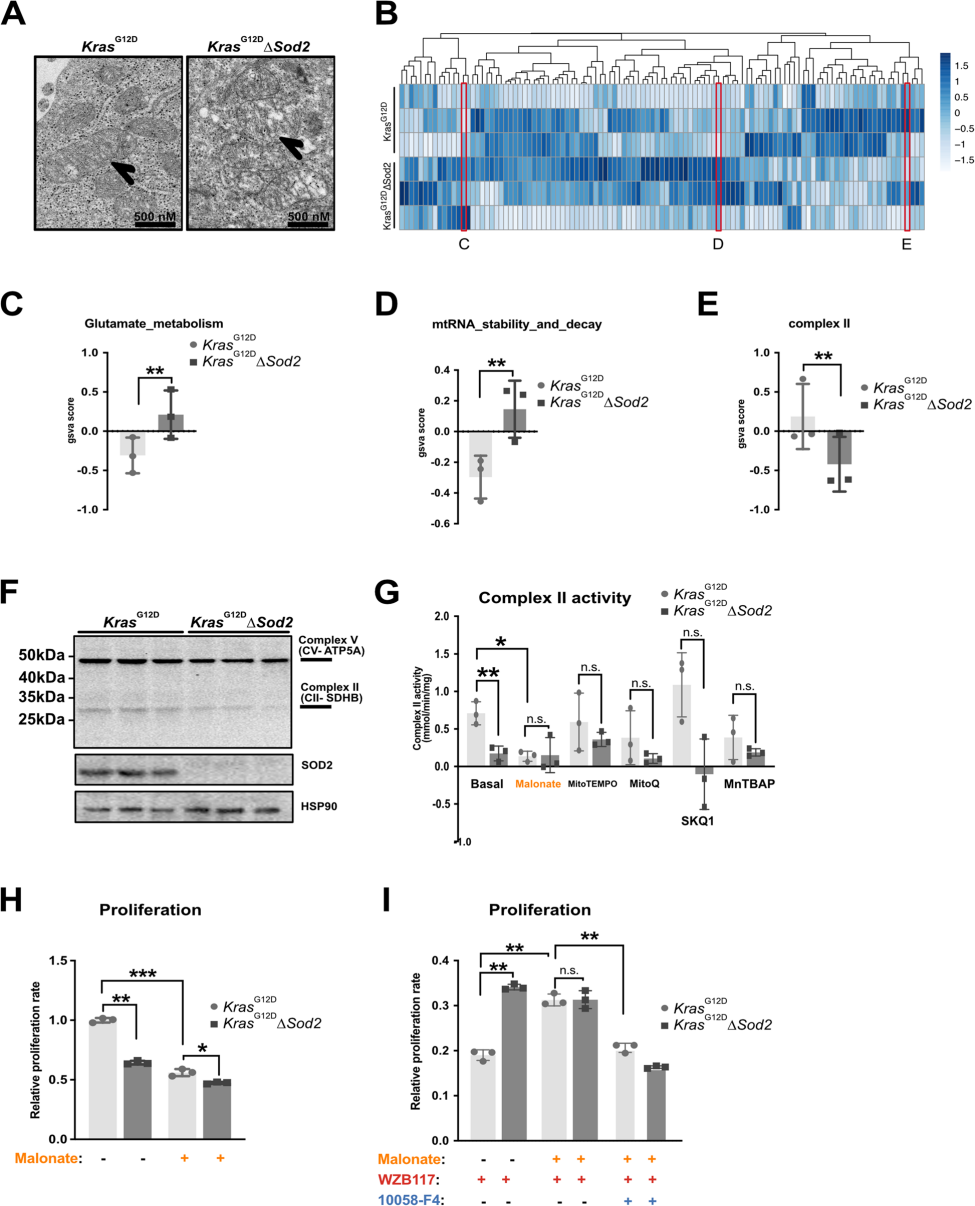
Complex II (SDH) inactivation phenocopies *Sod*2-deficiency. **A** Representative TEM images showing mitochondria from *Kras*^G12D^ control and *Kras*^G12D^∆*Sod2* cell lines. **B** Heatmap of enrichment scores for mitochondrial pathways in RNA-Seq data from 3 *Kras*^G12D^ and 3 *Kras*^G12D^*∆Sod2* cells, significantly different pathways are designated by "**C", "D", "E"** and the individual enrichment scores for these are shown in **C**, **D** and **E**. **F** Western blot for mitochondrial complexes in 3 *Kras*^G12D^ control cell lines and 3 *Kras*^G12D^∆*Sod2* cell lines **G** Complex II activity in 3 *Kras*^G12D^ control cell lines and 3 *Kras*^G12D^∆*Sod2* cell lines after 24 h of basal culture, 10 mM Malonate, 50 µM MitoTEMPO, 1 µM MitoQ, 10 µM SKQ1, and 100 µM MnTBAP **H** Relative proliferation rates of 3 *Kras*^G12D^ and 3 *Kras*^G12D^∆*Sod2* cancer cell lines after 72 h of basal culture and 10 mM Malonate. **I** Relative proliferation rates of 3 *Kras*^G12D^ and 3 *Kras*.^G12D^∆*Sod2* cancer cell lines after 72 h of 30 µM WZB117 (Glut1 inhibition), in combination with 10 mM Malonate and 60 µM 10058-F4, as indicated. Error bars are SD, p-value was calculated using Student’s t-test for paired samples. *, *p* < 0.05, **, *p* < 0.01, ***, *p* < 0.001

Given the significant changes in mitochondrial respiration, we performed transmission electron microscopy (TEM) to examine mitochondrial morphology in *Sod2*-deficient and control cells (Fig. 4A). Previous studies have shown that oxidative stress leads to cristae swelling [24]. Consistent with this, *Sod2*-deficient cells displayed a significantly higher number of mitochondria with a translucent matrix and enlarged, ballooned, or rounded cristae structures (for complete classification, see Fig. S2A). To further investigate the molecular basis of these mitochondrial changes, we conducted geneset enrichment analysis using MitoCarta3.0, a curated database of mitochondrial pathway genes [25] (Fig. 4B). Interestingly, only three pathways showed significant differences between *Sod2*-deficient and control cells. Genes involved in glutamate metabolism were upregulated (Fig. 4C). Genes related to mitochondrial tRNA stability and decay were upregulated (Fig. 4D). Complex II components were downregulated in *Sod2*-deficient cells (Fig. 4E). Western blot analysis of the electron transport chain (ETC) complex proteins (Fig. 4F) confirmed a strong reduction in the expression of complex II subunit SDHB in *Sod2*-deficient cells. To assess whether this reduction could contribute to the *Sod2*-deficiency phenotype, we used malonate, a well-established reversible inhibitor of complex II activity [26]. We found that complex II activity was significantly reduced in *Sod2*-deficient cells and malonate treatment lowered complex II activity in control cells to levels comparable to those seen in *Sod2*-deficient cells (Fig. 4G). To assess whether oxidative stress contributes to this reduction, cells were treated with the mitochondrial antioxidants MitoTEMPO, MitoQ, SKQ1, or MnTBAP. Following treatment with any of these antioxidants, the difference in complex II activity between control and *Sod2*-deficient cells was no longer significant, although complex II activity remained markedly reduced in *Sod2*-deficient cells and was only marginally increased by MitoTEMPO.

When cultured in the presence of malonate, control cell proliferation was significantly reduced to a rate similar to that of untreated *Sod2*-deficient cells (Fig. 4H). Additionally, malonate further reduced the proliferation of *Sod2*-deficient cells, suggestive of additional effects beyond complex II inhibition. To determine whether complex II inhibition contributes to the increased tolerance to Glut1 inhibition, we treated both *Sod2*-deficient and control cells with malonate, the Glut1 inhibitor WZB117, and Myc inhibitor 10058_F4. Notably, malonate treated control cells exhibited increased tolerance to Glut1 inhibition, mirroring the phenotype of *Sod2*-deficient cells. Interestingly, this improved tolerance to Glut1 inhibition after malonate addition was abrogated with Myc was inhibited (Fig. 4I). These findings demonstrate that complex II inactivation through SDH inhibition in control cells closely mimics the phenotype of *Sod2*-deficient cells, suggesting a crucial role for mitochondrial respiration and Myc signaling in mediating glucose metabolism adaptions in pancreatic cancer cells.

### Myc-dependent regulation of mitochondrial metabolism in *Sod2* deficient and complex II inhibited cells

**Fig. 5:**
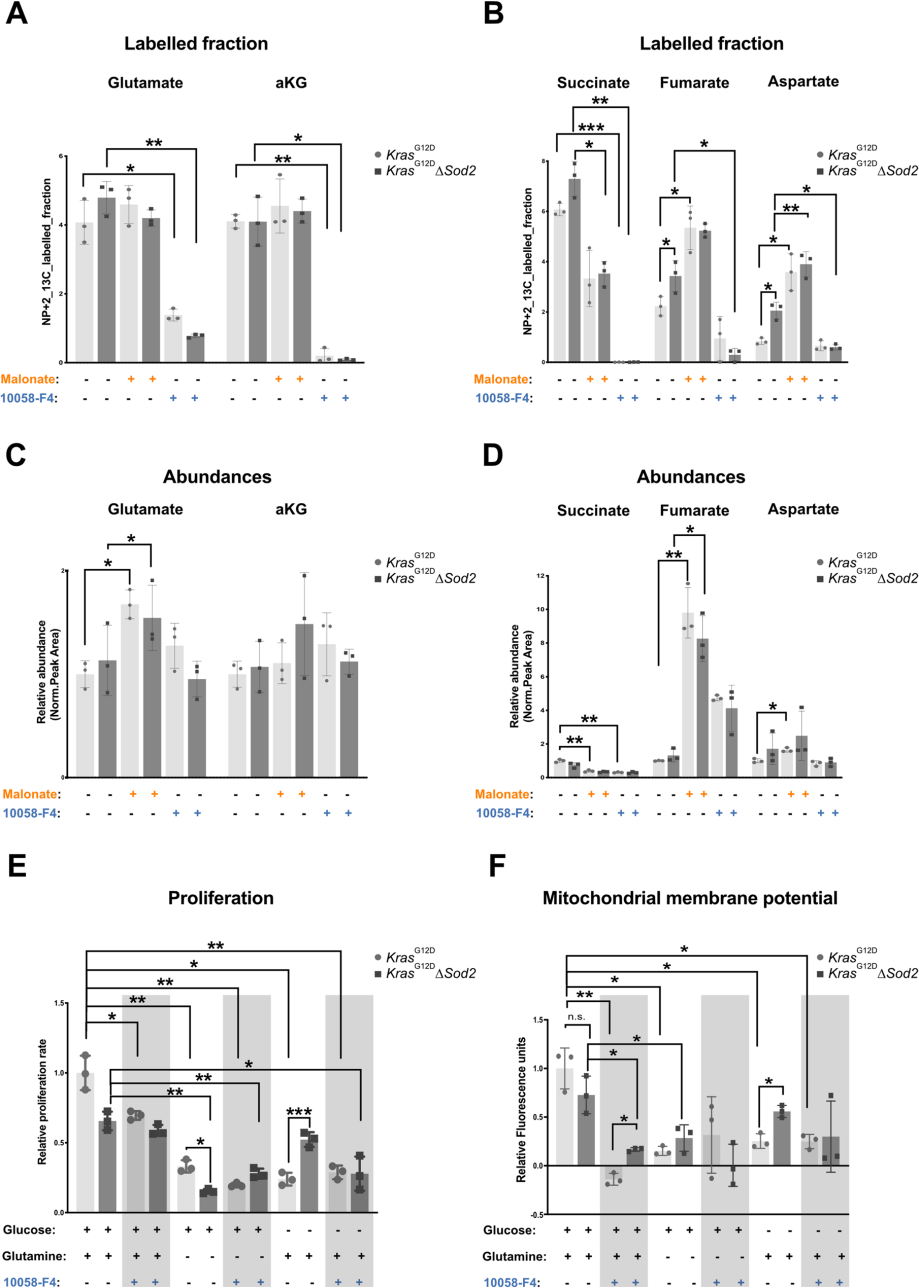
Mitochondrial metabolism in *Sod2*-deficient and Complex II inactivated cells is Myc-mediated. NP + 2 labeled fraction of metabolites after treatments with 24 h of 10 mM Malonate and 60 µM 10058-F4, as indicated, in 3 Kras^G12D^ control cell lines and 3 *Kras*^G12D^∆*Sod2* cell lines after ^13^C incorporation TCA cycle metabolites, **A** Glutamate and alpha-ketoglutarate (aKG), **B** Succinate, Fumarate, and Aspartate. Relative abundances of metabolites after treatments with 24 h of 10 mM Malonate and 60 µM 10058-F4, as indicated, in 3 Kras^G12D^ control cell lines and 3 *Kras*^G12D^∆*Sod2* cell lines after ^13^C incorporation TCA cycle metabolites, **C** Glutamate and alpha-ketoglutarate (aKG), **D** Succinate, Fumarate, and Aspartate. **E** Relative proliferation rates of 3 *Kras*^G12D^ and 3 *Kras*^G12D^∆*Sod2* cancer cell lines after 72 h of basal culture, Glucose-free medium (4 mM Glutamine), and Glutamine-free medium (25 mM Glucose), in combination with 60 µM 10058-F4, as indicated. **F** Mitochondrial membrane potential measured using TMRE dye from 3 *Kras*^G12D^ and 3 *Kras*.^G12D^*∆ Sod2* cells after 24 h of basal culture, Glucose-free medium (4 mM Glutamine), and Glutamine-free medium (25 mM Glucose), in combination with 60 µM 10058-F4, as indicated. Error bars are SD, *p*-value was calculated using Student’s t-test for paired samples. *, *p* < 0.05, **, *p* < 0.01, ***, *p* < 0.001

To gain deeper insight into the metabolic alterations caused by *Sod2* deficiency, complex II inactivation, and Myc inhibition, we performed ^13^C_6_ glucose tracing under basal conditions. By analyzing labelling patterns and metabolite abundances, we examined NP + 3 isotopologues (representing 3-carbon contribution from ^13^C_6_ glucose) for glycolytic metabolites and NP + 2 isotopologues (representing 2-carbon contribution by ^13^C_6_ glucose) for TCA cycle metabolites, providing a relative measure of pathway activity. Compared to control cells, *Sod2*-deficient cells exhibited a significant reduction in ^13^C-labeled pyruvate and lactate (NP + 3) (Fig. S3A), despite showing no significant differences in the overall abundances of these metabolites (Fig. S3B). Interestingly, malonate treatment markedly increased NP + 3 labeled pyruvate in both control and *Sod2*-deficient cells, whereas Myc inhibition significantly increased total pyruvate and lactate levels (Fig. S3B). PDH activity was unchanged between control and Sod2-deficient cells (Fig. S3C). In line with the previously observed Myc dependent increase in mitochondrial respiration, Myc inhibition significantly reduced the NP + 2 labelled fraction of key TCA cycle metabolites (glutamate, alpha-ketoglutarate (aKG), succinate, fumarate, and aspartate) (Fig. 5A, B). Notably, *Sod2*-deficient cells exhibited a significant increase in NP + 2 labeled succinate, fumarate, and aspartate compared to control cells, indicating enhanced oxidative metabolism following *Sod2* depletion (Fig. 5B). Furthermore, complex II inhibition with malonate increased NP + 2 labelled fumarate and aspartate in both control and *Sod2*-deficient cells, reinforcing the idea that malonate mimics the metabolic effects of *Sod2* deficiency. Additionally, malonate treatment led to a marked increase in NP + 2 labelled glutamate, fumarate, and aspartate, which was reflected in their overall metabolic abundances (Fig. 5C & D). These findings highlight that mitochondrial metabolism in *Sod2*-deficient and complex II inhibited cells are strongly Myc dependent, with Myc playing a central role in regulating oxidative metabolism and metabolic plasticity in pancreatic cancer cells.

With ATP production remaining comparable between control and *Sod2-*deficient cells (Fig. 2D), and the increased labelling and abundance of aspartate observed in *Sod2*-deficient cells (Fig. 5B, D), we propose that aspartate biosynthesis plays a crucial role in mitochondrial respiration in the absence of *Sod2*. To determine whether Myc activity is essential for aspartate metabolism, control cells and *Sod2*-deficient cells were treated with aspartate, WZB117, and 10058_F4. Cells supplemented with aspartate exhibited significantly increased tolerance to Glut1 inhibition compared to their untreated counterparts. However, this improved tolerance to Glut1 inhibition compared to their untreated counterparts. However, this aspartate-induced tolerance to Glut1 inhibition was abolished upon Myc inhibition (Fig. S3D).

Given the upregulation of genes associated with glutamine metabolism (Fig. 4C) and the increased oxidative labeling of mitochondrial TCA cycle metabolites (Fig. 5B) in *Sod2*-deficient cell lines, we further investigated the role of glutamine metabolism in cell proliferation. To this end, control and *Sod2*-deficient cells were cultured in glutamine-free medium or glucose-free medium in combination with 10,058-F4 (Fig. 5E). *Sod2*-deficient cells exhibited a significant reduction in proliferation when glutamine was depleted in glucose-containing medium. Interestingly, *Sod2*-deficient cells displayed a markedly increased tolerance to glucose deprivation when supplemented with glutamine. However, this glutamine-mediated resistance to glucose deprivation was completely lost upon Myc inhibition.

To test whether these metabolic differences were associated with altered mitochondrial function, we measured the mitochondrial membrane potential (Fig. 5F). Under basal conditions, *Sod2*-deficient cells exhibited a slightly reduced membrane potential compared to controls, although this difference did not reach significance. Myc inhibition markedly decreased the membrane potential in both groups. However, the residual potential remained significantly higher in *Sod2*-deficient cells. In glutamine-free, glucose-containing medium, the membrane potential was uniformly low and not significantly different between genotypes, irrespective of Myc inhibition. Upon glutamine supplementation, the membrane potential increased and was significantly higher in *Sod2*-deficient cells, an effect that was abrogated by Myc inhibition.

These findings highlight the critical role of Myc in supporting metabolic flexibility, allowing *Sod2*-deficient cells to adapt to nutrient stress by enhancing aspartate and glutamine metabolism.

### *Sod2*-dependent metabolism and myc-activation are mediated by peroxynitrite

**Fig. 6:**
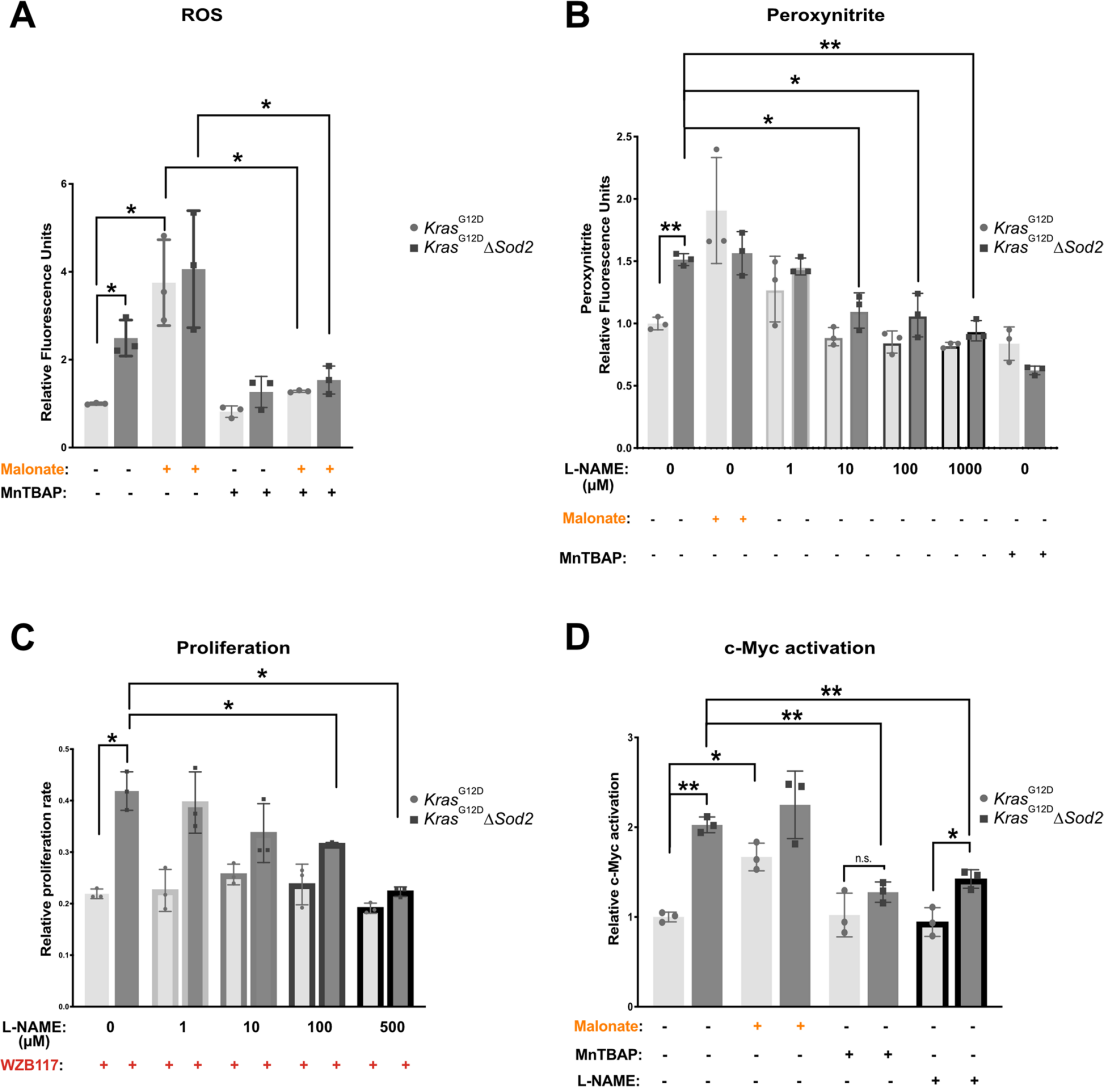
*Sod2*-dependent metabolism and Myc-activation are mediated by peroxynitrite. **A** ROS levels of 3 *Kras*^G12D^ control cell lines and 3 *Kras*^G12D^∆*Sod2* cell lines after 24 h of basal culture, 10 mM Malonate, and in combination with 100 µM MnTBAP, as indicated. **B** Peroxynitrite levels of 3 *Kras*^G12D^ control cell lines and 3 *Kras*^G12D^∆*Sod2* cell lines after 2-h of basal culture, 10 mM Malonate, different doses of L-NAME (0 µM – 1000 µM), and 100 µM MnTBAP, as indicated. **C** Relative proliferation rates of 3 *Kras*^G12D^ and 3 *Kras*^G12D^∆*Sod2* cancer cell lines after 72 h of 30 µM WZB117 (Glut1 inhibition) in combination with different doses of L-NAME, as indicated. **D** Quantification of Myc transcriptional activation from 3 *Kras*^G12D^ and 3 *Kras*.^G12D^∆*Sod2* cells after 24 h of basal culture, 10 mM Malonate, 100 µM MnTBAP, and 1 mM L-NAME. Error bars are SD, p-value was calculated using Student’s t-test for paired samples. *, *p* < 0.05, **, *p* < 0.01

Consistent with the elevated ROS levels in *Sod2*-deficient cells and their reduction upon MnTBAP treatment (Fig. 1C), we observed that malonate treatment increased ROS levels in both control and *Sod2*-deficient cells. This ROS induction was abolished with MnTBAP (Fig. 6A). Given the elevated superoxide levels in S*od2*-deficient cells, we investigated whether this oxidative stress contributes to increased Myc activation. Superoxide is known to react with nitric oxide, leading to the formation of peroxynitrite [27]. Accordingly, we detected a significant increase in peroxynitrite levels in both *Sod2*-deficient cells and in malonate treated control cells (Fig. 6B). Notably, both MnTBAP treatment and nitric oxide synthetase inhibition with L-NAME significantly reduced peroxynitrite levels. Furthermore, the increased tolerance of *Sod2*-deficient cells to glucose deprivation (Fig. 2K), was abolished by increasing concentrations of L-NAME (Fig. 6C), highlighting a role for peroxynitrite in metabolic adaptation. Previous studies have shown that oxidative modifications induced by peroxynitrite can enhance Myc stabilization and activation [28], suggesting a direct link between oxidative stress and Myc regulation.

The increased tolerance of malonate-treated control cells to Glut1 inhibition (F 4g. 4I), along with enhanced labelling and abundances of fumarate and aspartate (Fig. 5B & D) suggest that malonate-mediated complex II inactivation may lead to increased Myc activation. We hypothesized that malonate-induced ROS production promotes Myc activation via peroxynitrite formation. To test this, we assessed Myc transcriptional activity in nuclear extracts of control and *Sod2*-deficient cells following treatment with malonate, MnTBAP, and L-NAME. As expected, complex II inactivation with malonate significantly increased Myc activation in control cells. Additionally, both L-NAME and MnTBAP significantly reduced Myc activation in *Sod2*-deficient cells (Fig. 6D).

These findings demonstrate that *Sod2*-deficiency or complex II inhibition induces a metabolic shift through peroxynitrite formation and Myc activation, promoting a glutamine-preferred, aspartate-producing metabolic state.

## Discussion

In our study, *Sod2* deletion led to increased ROS levels, complex II inhibition, and elevated peroxynitrite formation. The resulting Myc activation and enhanced mitochondrial respiration increased glutamine utilization and dependence, conferring resistance to glucose deprivation.

The increased Myc activity was attenuated either by reducing superoxide levels with MnTBAP or by inhibiting peroxynitrite formation with L-NAME.

Complex II contains iron-sulfur clusters, which are highly sensitive to oxidative stress [29]. We propose that oxidation of these clusters underlies the reduction in complex II levels and activity in *Sod2*-deficient cells. To explore whether complex II inhibition directly contributes to the observed effects, we treated control cells with malonate, a well-established competitive inhibitor of complex II. Malonate treatment recapitulated several key features of *Sod2* deletion, including reduced complex II activity, decreased proliferation, increased Myc-dependent glucose deprivation tolerance, and elevated increased ROS and peroxynitrite levels. Notably, Malonate had little effect on proliferation or complex II activity in *Sod2*-deficient cells, suggesting a similar functional mechanism.

However, significant differences emerged in ^13^C labeling experiments. Malonate treatment led to a greater increase in labeled fumarate and aspartate, as well as a higher overall fumarate abundance compared to *Sod2*-deletion alone. Additionally, labeled succinate levels decreased upon malonate exposure. These findings are intriguing, as succinate dehydrogenase (SDH) typically catalyzes the oxidation of succinate to fumarate. One possible explanation is that malonate promotes a reverse SDH reaction, where SDH reduces fumarate to succinate, a phenomenon previously reported under hypoxic conditions [30].

The effect of malonate on ROS and peroxynitrite appears to be context-dependent, as previous studies have reported both decreased [31] and increased ROS production [32–34] following malonate treatment.

Although multiple studies have shown increased ROS levels following MYC induction [35, 36], there is also evidence of the reverse relationship, were ROS can induce MYC expression. For instance, H_2_O_2_ has been shown to upregulate MYC, while antioxidants inhibited MYC expression [37]. The impact of MYC on mitochondrial respiration is multifaceted: studies have reported both MYC-driven respiratory induction [38] and a decrease in oxygen consumption Rate (OCR) upon Myc inhibition [39].

Several studies have examined the role of MnSOD in pancreatic cancer, with most reporting that high MnSOD expression reduces proliferation [40–42]. However, a recent study found results similar to ours [43]. These contradictory findings may stem from differences in cell line models or the overexpression of MnSOD via adenoviral transfer which may exceed the physiological levels. Our findings provide key insights into the association between low SOD2 expression and a survival advantage in two pancreatic cancer cohorts (TCGA, ICGC). Consistent with Mehmetoglu-Gurbuz et al., we propose that SOD2 inhibition could represent a promising therapeutic strategy for PDAC patients.

## Conclusion

SOD2 deficiency triggers metabolic adaptation in cancer cells by increasing ROS and peroxynitrite. This results in increased Myc activity, enhanced mitochondrial respiration and improved survival under glucose deprivation. Similarly, malonate treatment mimics effects of SOD2 deficiency on ROS, peroxynitrite, and survival under glucose deprivation. In conclusion, our findings highlight the role of SOD2 in tumor progression and metabolic adaptation.

## Supplementary Information

Supplementary Material 1. Supplementary Figure S1. A Kaplan-Meier plot for overall survival based on high or low expression of SOD2 in patients from the ICGC PACA-AU cohort. Below the number of patients at risk. B Kaplan-Meier plot for overall survival based on high or low expression of SOD2 in patients from the TCGA PAAD cohort. Below the number of patients at risk. C Representative Sanger sequencing chromatograms of Sod2 loci from 3 KrasG12D control cell linesand 3 KrasG12D∆Sod2 cell linesshowing the presence of alterations induced by CRISPR-Cas9-mediated Sod2 deletion. D RT-PCR analysis of Sod2 mRNA expression from 3 KrasG12D control cell linesand 3 KrasG12D∆Sod2 cell lines. E GSH/GSSG ratio in control and KrasG12D∆Sod2 cell lines. Morphology of 3 KrasG12D control cell linesand their respective Sod2 deficient clones, by light microscopy. Scale bar, 100 µm. Error bars are SD, all p-values were calculated using Student’s t-test for paired samples. *, *p*< 0.05.

Supplementary Material 2. Supplementary Figure S2. A Mitochondria were classified into 4 groups (class 1, class 2, class 3, and class 4. Class 1 mitochondria are characterized by well-defined features such as an ellipsoid structure, with intact outer membranes, structured matrix, and abundant parallel cristae. Class 2 includes mitochondria that display intact outer membrane and contain translucent areas within the matrix. Class 3 includes mitochondria that show ballooning of cristae structures in addition to translucent matrix. Class 4 includes mitochondria that have poorly defined features. Quantification of mitochondrial classes present from 3 KrasG12D and 3 KrasG12D∆Sod2 cancer cell lines. Error bars are SD, p-value was calculated using Student’s t-test for paired samples. *, *p*< 0.05.

Supplementary Material 3. Supplementary Figure S3. A NP+3 labeled fraction of pyruvate and lactate after treatments with 24 hours of 10 mM Malonate and 60 µM 10058-F4, as indicated, in 3 KrasG12D control cell lines and 3 KrasG12D∆Sod2 cell lines after 13 C incorporation TCA cycle. B Relative abundances of pyruvate and lactate after treatments with 24 hours of 10 mM Malonate and 60 µM 10058-F4, as indicated, in 3 KrasG12D control cell lines and 3 KrasG12D∆Sod2 cell lines after 13 C incorporation. C PDH activity measured from 3 KrasG12D and 3 KrasG12D∆ Sod2 cells. D Relative proliferation rate of 3 KrasG12D and 3 KrasG12D∆Sod2 cancer cell lines after 72 hours of 30 µM WZB117, in combination with 10 mM Aspartate and 60 µM 10058-F4, as indicated. Error bars are SD, p-value was calculated using Student’s t-test for paired samples. *, *p*< 0.05, **, *p*<0.01, ***, *p*< 0.001.

Supplementary Material 4. Supplementary Figure S4. Raw image files of blots used in the manuscript for Figures 1 A, 1D, 2B, and 4F.

Supplementary Material 5. Supplementary Table S1. Association of genes with annotation GO:0016209with outcomes of pancreatic cancer patients from PACA-AU and TCGA-PAAD. "outcome" = outcome in cohort with high expression, NA not available.

Supplementary Material 6. Supplementary Table S2. Results of gsva analysis of Msigdb hallmark genesets between control KrasG12D and KrasG12D∆Sod2 cell lines.

## Data Availability

The data generated in this study are available upon request from the corresponding author. RNA-seq data have been deposited in the EMBL-EBI BioStudies database under accession number E-MTAB-16320.

## Abbreviations

SOD2/MnSOD: Superoxide dismutase2/Manganese-dependent superoxide dismutase
ROS: Reactive oxygen species
PDAC: Pancreatic ductal adenocarcinoma
SDH: Succinate dehydrogenase
c-MYC: Cellular myelocytomatosis
GLUT1: Glucose transporter type 1
AMPK: AMP-activated protein kinase
HSP90: Heat shock protein 90
CRISPR: Clustered Regularly Interspaced Short Palindromic Repeats
Cas9: CRISPR-associated protein 9
LC-MS: Liquid chromatography-mass spectrometry
DMEM: Dulbecco’s Modified Eagle Medium
ATP: Adenosine Triphosphate
L-NAME: L-N^G^-nitro arginine methyl ester
MnTBAP: Mn(III)tetrakis(4-benzoic acid)porphyrin chloride
