# Correction: Mitochondrial superoxide dismutase controls metabolic plasticity in pancreatic cancer

**DOI:** 10.1186/s12964-026-02750-1

**Published:** 2026-02-20

**Authors:** Sankaranarayanan Ramasubramanian, Rupert Öllinger, Carola Eberhagen, Hans Zischka, Roland M. Schmid, Henrik Einwächter

**Affiliations:** 1 Department of Medicine 2, School of Medicine and Health, Technical University of Munich, Ismaninger Straße 22, Munich, 81675 Germany; 2 Institute of Molecular Oncology and Functional Genomics, School of Medicine and Health, Technical University of Munich, Munich, Germany; 3 Institute of Molecular Toxicology and Pharmacology, Helmholtz Center Munich, German Research Center for Environmental Health, Ingolstädter Landstraße 1, Neuherberg, 85764 Germany; 4 Institute of Toxicology and Environmental Hygiene, School of Medicine and Health, Technical University Munich, Biedersteinerstraße 29, Munich, 80802 Germany

**Correction: Ramasubramanian et al. Cell Communication and Signaling 23, 524 (2025)**

**https://doi.org/10.1186/s12964-025-02555-8**

Following publication of the original article [1], authors requested to insert two reference details (16. Bailey P, Chang DK, Nones K, Johns AL, Patch A-M, Gingras M-C, et al. Genomic analyses identify molecular subtypes of pancreatic cancer. Nature 2016;531:47–52 and 17. Cancer Genome Atlas Research Network. Comprehensive genomic characterization defines human glioblastoma genes and core pathways. Nature 2008;455:1061–1068) which were not added by the correction team during the revision process which led to the incorrect linking of the in-text citations.

The original article [1] has been corrected.
