# Supplementary figures and images for: Mitochondrial superoxide dismutase controls metabolic plasticity in pancreatic cancer
Source: Cell Commun Signal. 2025 Dec 6;23:524. doi: 10.1186/s12964-025-02555-8 (PMC12690781; doi:10.1186/s12964-025-02555-8)

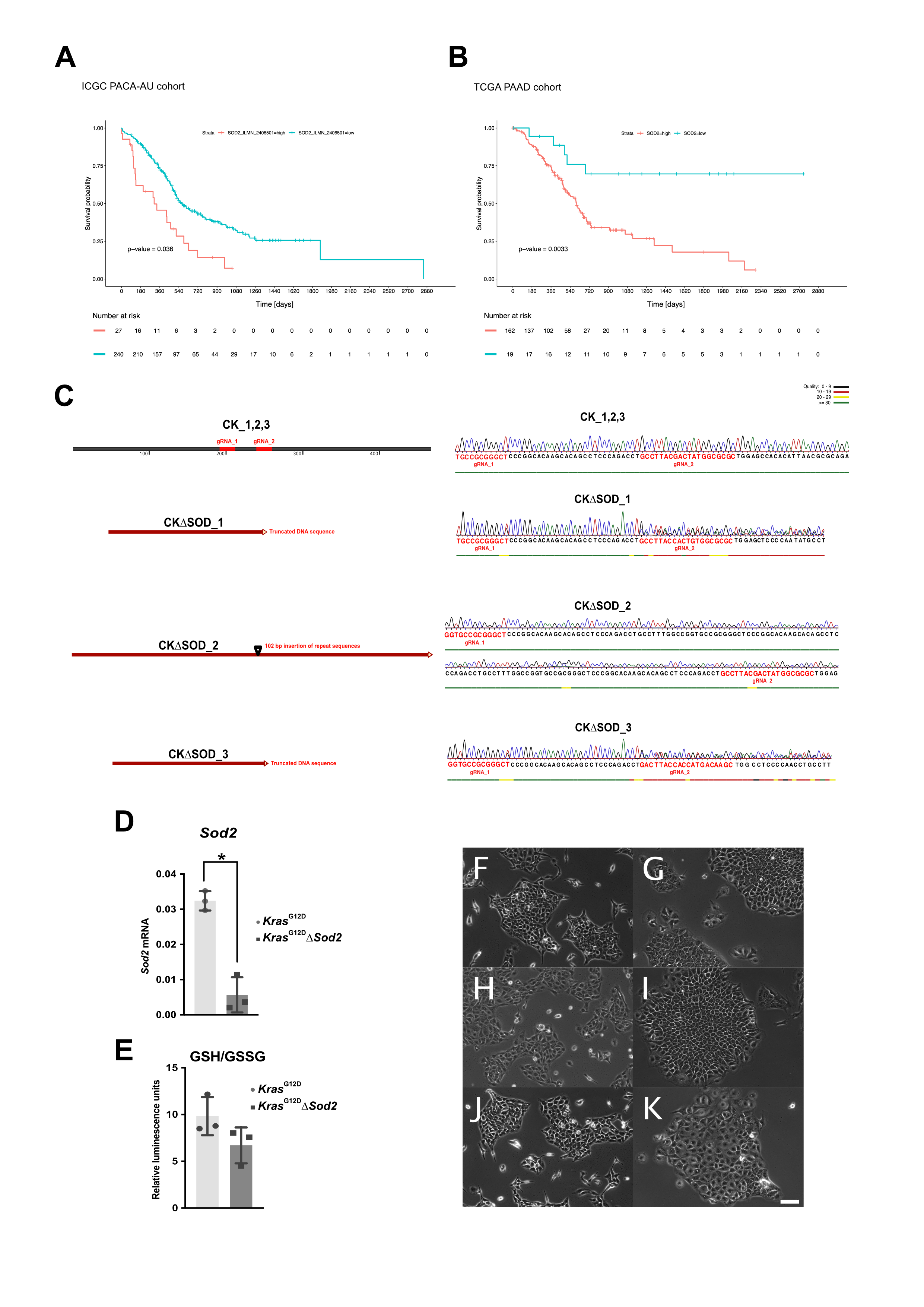

Supplement: Supplementary file 1 — Supplementary Material 1. Supplementary Figure S1. A Kaplan-Meier plot for overall survival based on high or low expression of SOD2 in patients from the ICGC PACA-AU cohort. Below the number of patients at risk. B Kaplan-Meier plot for overall survival based on high or low expression of SOD2 in patients from the TCGA PAAD cohort. Below the number of patients at risk. C Representative Sanger sequencing chromatograms of Sod2 loci from 3 KrasG12D control cell linesand 3 KrasG12D∆Sod2 cell linesshowing the presence of alterations induced by CRISPR-Cas9-mediated Sod2 deletion. D RT-PCR analysis of Sod2 mRNA expression from 3 KrasG12D control cell linesand 3 KrasG12D∆Sod2 cell lines. E GSH/GSSG ratio in control and KrasG12D∆Sod2 cell lines. Morphology of 3 KrasG12D control cell linesand their respective Sod2 deficient clones, by light microscopy. Scale bar, 100 µm. Error bars are SD, all p-values were calculated using Student’s t-test for paired samples. *, p< 0.05. [file 12964_2025_2555_MOESM1_ESM.tiff]

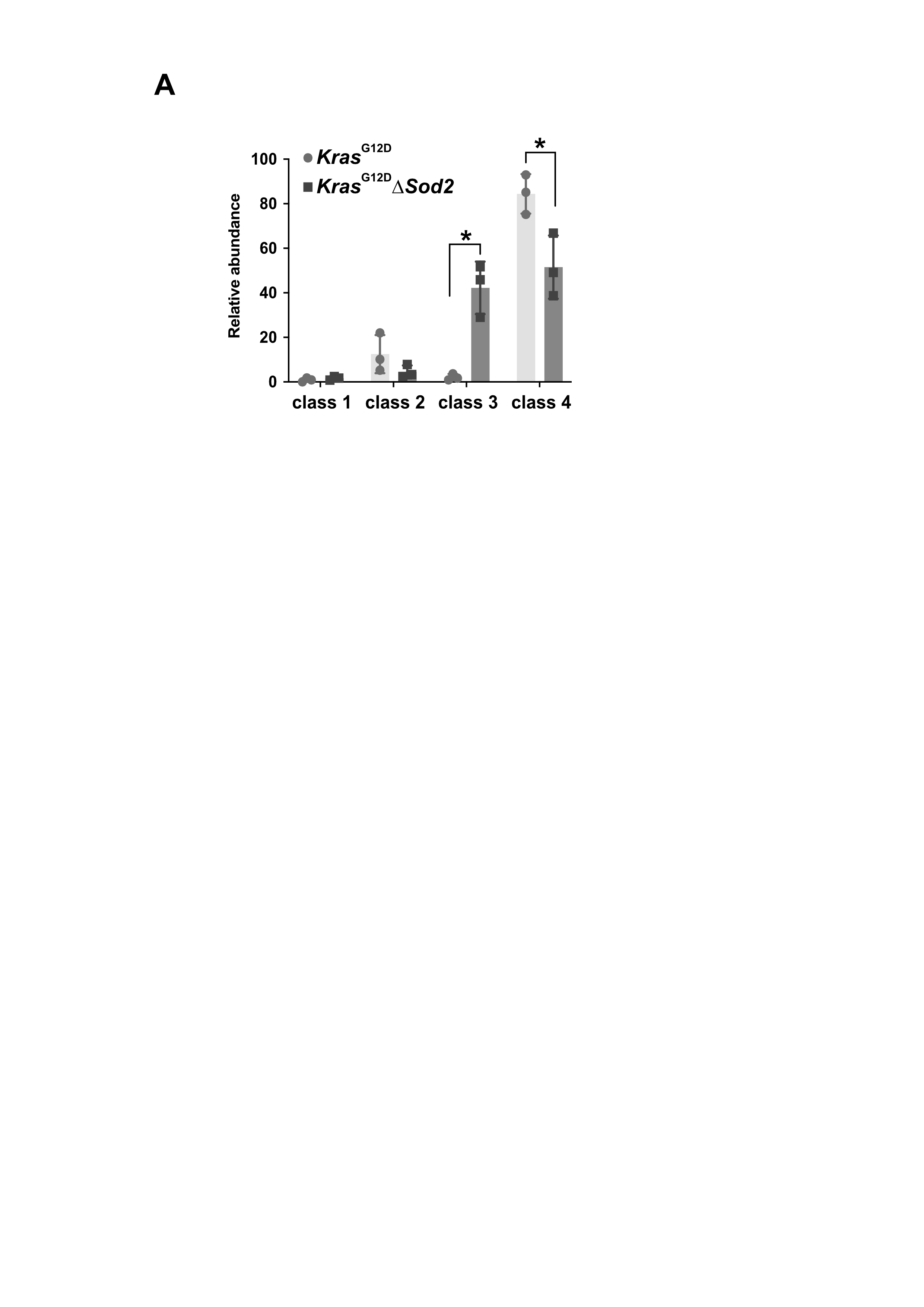

Supplement: Supplementary file 2 — Supplementary Material 2. Supplementary Figure S2. A Mitochondria were classified into 4 groups (class 1, class 2, class 3, and class 4. Class 1 mitochondria are characterized by well-defined features such as an ellipsoid structure, with intact outer membranes, structured matrix, and abundant parallel cristae. Class 2 includes mitochondria that display intact outer membrane and contain translucent areas within the matrix. Class 3 includes mitochondria that show ballooning of cristae structures in addition to translucent matrix. Class 4 includes mitochondria that have poorly defined features. Quantification of mitochondrial classes present from 3 KrasG12D and 3 KrasG12D∆Sod2 cancer cell lines. Error bars are SD, p-value was calculated using Student’s t-test for paired samples. *, p< 0.05. [file 12964_2025_2555_MOESM2_ESM.tiff]

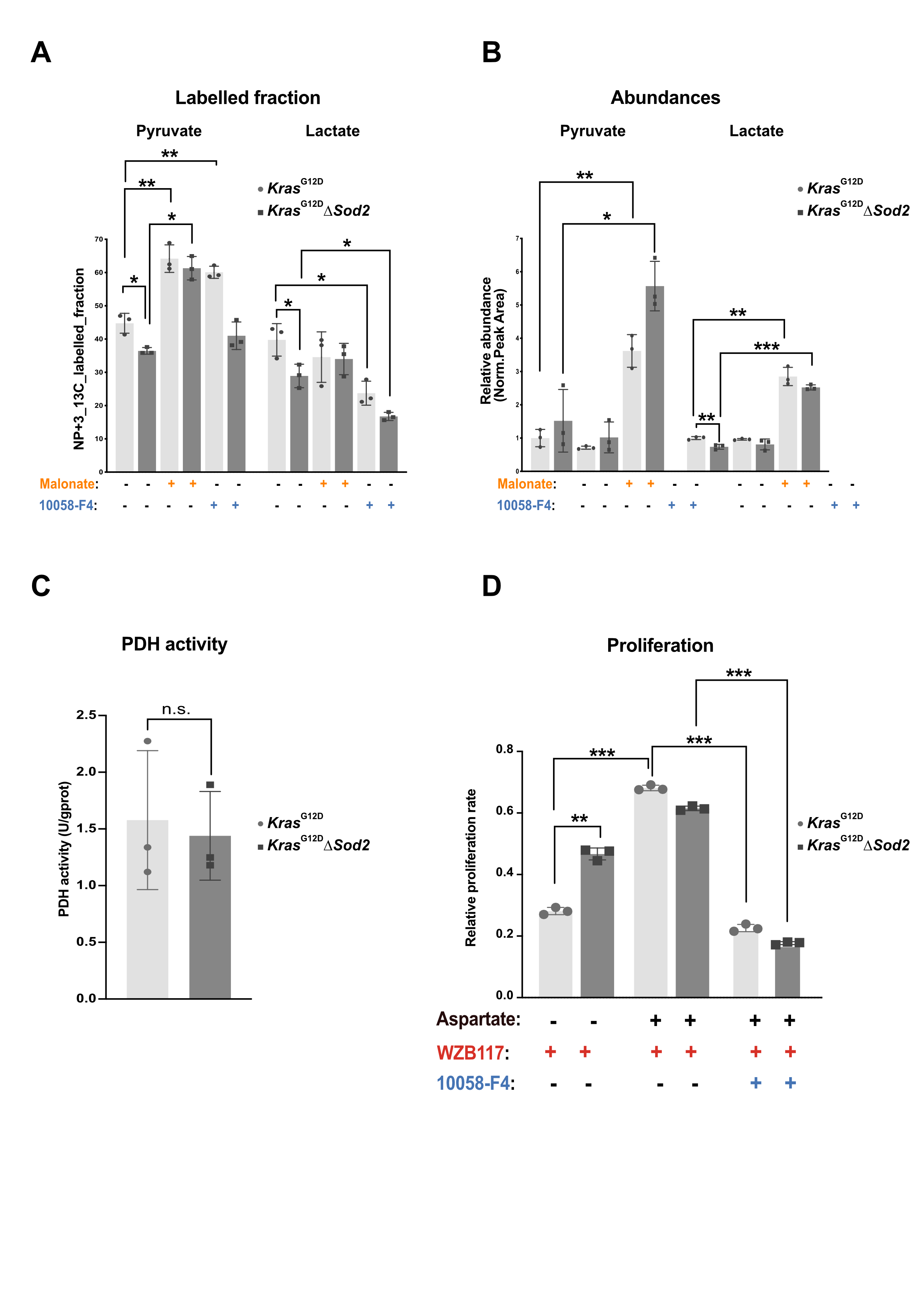

Supplement: Supplementary file 3 — Supplementary Material 3. Supplementary Figure S3. A NP+3 labeled fraction of pyruvate and lactate after treatments with 24 hours of 10 mM Malonate and 60 µM 10058-F4, as indicated, in 3 KrasG12D control cell lines and 3 KrasG12D∆Sod2 cell lines after 13 C incorporation TCA cycle. B Relative abundances of pyruvate and lactate after treatments with 24 hours of 10 mM Malonate and 60 µM 10058-F4, as indicated, in 3 KrasG12D control cell lines and 3 KrasG12D∆Sod2 cell lines after 13 C incorporation. C PDH activity measured from 3 KrasG12D and 3 KrasG12D∆ Sod2 cells. D Relative proliferation rate of 3 KrasG12D and 3 KrasG12D∆Sod2 cancer cell lines after 72 hours of 30 µM WZB117, in combination with 10 mM Aspartate and 60 µM 10058-F4, as indicated. Error bars are SD, p-value was calculated using Student’s t-test for paired samples. *, p< 0.05, **, p<0.01, ***, p< 0.001. [file 12964_2025_2555_MOESM3_ESM.tiff]

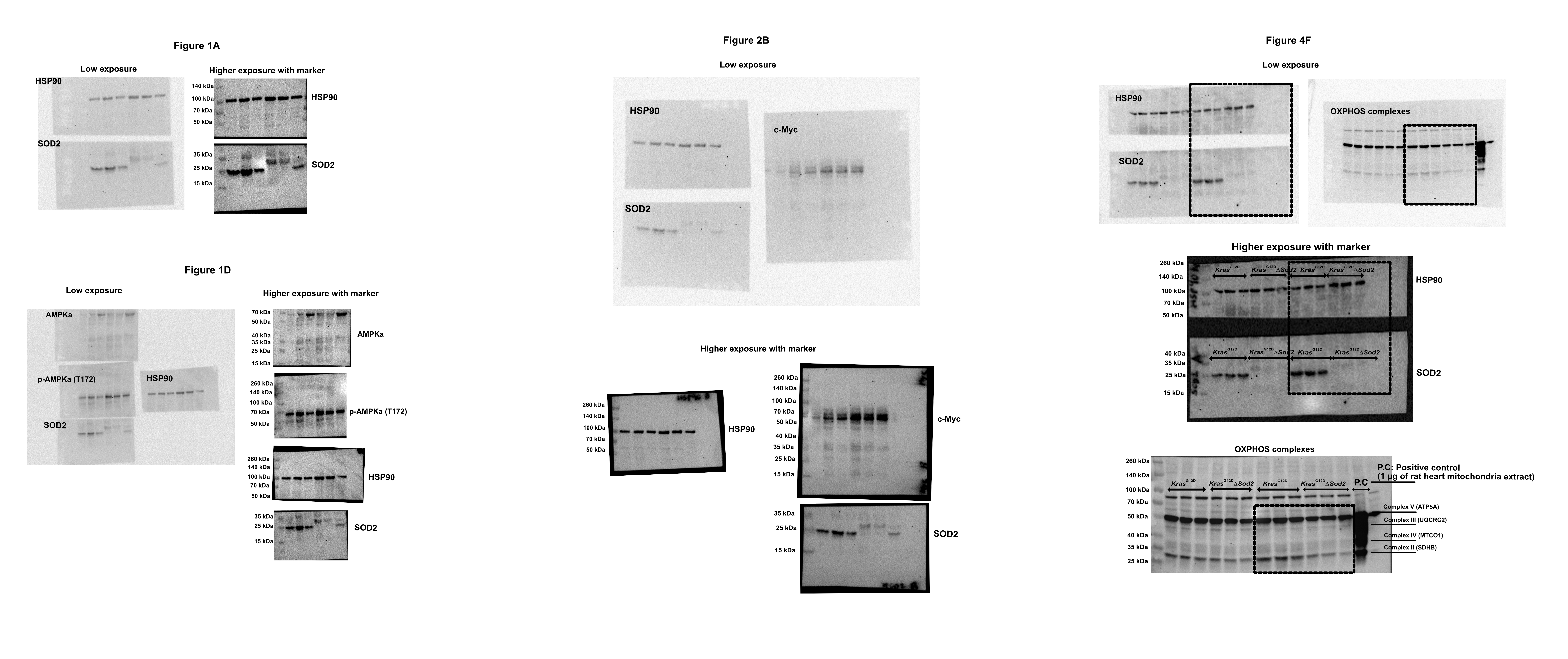

Supplement: Supplementary file 4 — Supplementary Material 4. Supplementary Figure S4. Raw image files of blots used in the manuscript for Figures 1 A, 1D, 2B, and 4F. [file 12964_2025_2555_MOESM4_ESM.tiff]
