# Supplementary material for: Mitochondrial superoxide dismutase controls metabolic plasticity in pancreatic cancer
Source: Cell Commun Signal. 2025 Dec 6;23:524. doi: 10.1186/s12964-025-02555-8 (PMC12690781; doi:10.1186/s12964-025-02555-8)
Supplement: Supplementary file 5 — Supplementary Material 5. Supplementary Table S1. Association of genes with annotation GO:0016209with outcomes of pancreatic cancer patients from PACA-AU and TCGA-PAAD. "outcome" = outcome in cohort with high expression, NA not available. [file 12964_2025_2555_MOESM5_ESM.pdf]

| PACA-AU | | | TCGA PAAD | | |
|---|---|---|---|---|---|
| probe | outcome | p-value | gene | outcome | p-value |
| APOM_ILMN_1731941 | better | 0.0001 | APOM | better | 0.2683 |
| LTC4S_ILMN_1668247 | better | 0.0002 | LTC4S | better | 0.0006 |
| CLIC2_ILMN_1795754 | better | 0.0002 | CLIC2 | better | 0.7603 |
| MGST1_ILMN_2355168 | worse | 0.0005 | MGST1 | worse | 0.0010 |
| PTGES_ILMN_1713829 | worse | 0.0012 | PTGES | worse | 0.0002 |
| SOD3_ILMN_1690034 | better | 0.0022 | SOD3 | better | 0.3482 |
| GSTK1_ILMN_1725241 | better | 0.0048 | GSTK1 | worse | 0.0483 |
| APOA4_ILMN_1675706 | better | 0.0058 | APOA4 | worse | 0.1801 |
| MGST1_ILMN_1781952 | worse | 0.0076 | MGST1 | worse | 0.0010 |
| SOD1_ILMN_1662438 | better | 0.0090 | SOD1 | better | 0.5419 |
| CP_ILMN_1813206 | worse | 0.0097 | CP | worse | 0.0214 |
| CAT_ILMN_2151739 | better | 0.0119 | CAT | worse | 0.1362 |
| NQO1_ILMN_2354953 | better | 0.0185 | NQO1 | worse | 0.0478 |
| TPO_ILMN_2357155 | better | 0.0244 | TPO | better | 0.5272 |
| GPX2_ILMN_2133205 | better | 0.0281 | GPX2 | worse | 0.4617 |
| GSTO2_ILMN_1740234 | worse | 0.0325 | GSTO2 | worse | 0.1181 |
| SOD2_ILMN_2406501 | worse | 0.0361 | SOD2 | worse | 0.0033 |
| TXNDC2_ILMN_1669460 | worse | 0.0377 | TXNDC2 | worse | 0.1706 |
| TP53INP1_ILMN_1714108 | better | 0.0387 | TP53INP1 | better | 0.1350 |
| TXNDC17_ILMN_1659437 | worse | 0.0445 | TXNDC17 | worse | 0.1634 |
| CYGB_ILMN_1758128 | better | 0.0560 | CYGB | better | 0.0602 |
| GSTT1_ILMN_1730054 | better | 0.0649 | GSTT1 | worse | 0.6624 |
| SOD2_ILMN_2336781 | worse | 0.0678 | SOD2 | worse | 0.0033 |
| PXDN_ILMN_2363658 | worse | 0.0683 | PXDN | worse | 0.0986 |
| CAT_ILMN_1651705 | better | 0.0718 | CAT | worse | 0.1362 |
| TXNRD2_ILMN_1669083 | worse | 0.0718 | TXNRD2 | better | 0.0378 |
| PRDX5_ILMN_2383975 | worse | 0.0749 | PRDX5 | worse | 0.4164 |
| HBB_ILMN_2100437 | better | 0.0751 | HBB | better | 0.6907 |
| MGST1_ILMN_1803033 | worse | 0.0786 | MGST1 | worse | 0.0010 |
| MT3_ILMN_1675947 | worse | 0.0795 | MT3 | better | 0.3020 |
| HBZ_ILMN_1713458 | better | 0.0834 | HBZ | better | 0.1014 |
| GSTZ1_ILMN_2381296 | worse | 0.0888 | GSTZ1 | better | 0.3781 |
| PTGS2_ILMN_2054297 | worse | 0.0892 | PTGS2 | worse | 0.0120 |
| GSTM2_ILMN_2201580 | better | 0.0911 | GSTM2 | better | 0.0205 |
| TXN_ILMN_2038776 | worse | 0.0979 | TXN | worse | 0.0085 |
| GSTO1_ILMN_2227573 | worse | 0.1067 | GSTO1 | worse | 0.2851 |
| HBA1_ILMN_3240144 | better | 0.1086 | HBA1 | better | 0.0137 |
| TXNRD1_ILMN_1717056 | worse | 0.1125 | TXNRD1 | worse | 0.0873 |
| GPX5_ILMN_2385410 | worse | 0.1209 | GPX5 | NA | NA |
| NXN_ILMN_1791226 | worse | 0.1218 | NXN | worse | 0.2751 |
| AMBP_ILMN_1695446 | better | 0.1239 | AMBP | worse | 0.9475 |
| MGST2_ILMN_1802027 | better | 0.1260 | MGST2 | worse | 0.3499 |
| PTGS2_ILMN_1677511 | worse | 0.1391 | PTGS2 | worse | 0.0120 |
| S100A9_ILMN_1750974 | worse | 0.1422 | S100A9 | worse | 0.0067 |
| GPX4_ILMN_2378952 | worse | 0.1470 | GPX4 | better | 0.0271 |
| PRDX1_ILMN_2366391 | better | 0.1550 | PRDX1 | worse | 0.0162 |
| DUOX1_ILMN_2404182 | worse | 0.1711 | DUOX1 | worse | 0.0160 |
| TXNRD2_ILMN_1657893 | worse | 0.1756 | TXNRD2 | better | 0.0378 |
| HBM_ILMN_2091454 | better | 0.1813 | HBM | better | 0.0225 |
| GSTA1_ILMN_1701831 | better | 0.2038 | GSTA1 | worse | 0.2765 |
| ALB_ILMN_1782939 | worse | 0.2266 | ALB | worse | 0.1134 |
| TXNDC2_ILMN_1658537 | worse | 0.2434 | TXNDC2 | worse | 0.1706 |
| PRDX3_ILMN_2395969 | worse | 0.2454 | PRDX3 | worse | 0.3079 |
| GSTA1_ILMN_2113470 | better | 0.2493 | GSTA1 | worse | 0.2765 |
| GSTA1_ILMN_3251497 | better | 0.2499 | GSTA1 | worse | 0.2765 |
| NQO1_ILMN_1720282 | better | 0.2609 | NQO1 | worse | 0.0478 |
| PXDNL_ILMN_1728481 | worse | 0.2640 | PXDNL | worse | 0.2752 |
| PRDX2_ILMN_1709584 | worse | 0.2646 | PRDX2 | better | 0.0001 |
| TP53INP1_ILMN_2214197 | better | 0.2704 | TP53INP1 | better | 0.1350 |
| PTGS1_ILMN_2339835 | better | 0.2716 | PTGS1 | worse | 0.5677 |
| LPO_ILMN_1670767 | worse | 0.2844 | LPO | better | 0.4590 |
| GSTM2_ILMN_1713162 | better | 0.2859 | GSTM2 | better | 0.0205 |
| SRXN1_ILMN_1804822 | worse | 0.2867 | SRXN1 | worse | 0.4121 |
| EPX_ILMN_1772631 | worse | 0.2922 | EPX | better | 0.0001 |
| GPX7_ILMN_1726030 | worse | 0.2930 | GPX7 | worse | 0.6532 |
| HBE1_ILMN_1651358 | better | 0.3050 | HBE1 | worse | 0.5400 |
| APOE_ILMN_1740938 | better | 0.3066 | APOE | better | 0.1728 |
| PRDX2_ILMN_2382829 | worse | 0.3068 | PRDX2 | better | 0.0001 |
| MB_ILMN_1666109 | worse | 0.3156 | MB | worse | 0.0083 |
| TXNDC2_ILMN_1653607 | worse | 0.3264 | TXNDC2 | worse | 0.1706 |
| PRDX3_ILMN_2395974 | worse | 0.3276 | PRDX3 | worse | 0.3079 |
| ALOX5AP_ILMN_1797875 | worse | 0.3424 | ALOX5AP | worse | 0.2082 |
| MGST3_ILMN_1751956 | better | 0.3448 | MGST3 | worse | 0.4753 |
| GPX3_ILMN_1726666 | better | 0.3483 | GPX3 | better | 0.0083 |
| HBD_ILMN_1815527 | better | 0.3642 | HBD | worse | 0.8304 |
| TXN_ILMN_1680314 | worse | 0.3869 | TXN | worse | 0.0085 |
| DUOX1_ILMN_1690289 | worse | 0.4002 | DUOX1 | worse | 0.0160 |
| DUOX2_ILMN_1786335 | worse | 0.4037 | DUOX2 | worse | 0.0968 |
| TXNRD1_ILMN_2324421 | worse | 0.4042 | TXNRD1 | worse | 0.0873 |
| PTGS1_ILMN_1665100 | worse | 0.4185 | PTGS1 | worse | 0.5677 |
| GSR_ILMN_2127416 | worse | 0.4247 | GSR | worse | 0.2172 |
| PRDX4_ILMN_2222234 | better | 0.4310 | PRDX4 | better | 0.0858 |
| PRDX2_ILMN_1767766 | worse | 0.4349 | PRDX2 | better | 0.0001 |
| HBG1_ILMN_1796678 | better | 0.4460 | HBG1 | worse | 0.6472 |
| PXDNL_ILMN_2158902 | worse | 0.4475 | PXDNL | worse | 0.2752 |
| PXDNL_ILMN_1746954 | better | 0.4500 | PXDNL | worse | 0.2752 |
| HBQ1_ILMN_1696183 | worse | 0.4552 | HBQ1 | better | 0.3726 |
| PRDX5_ILMN_1815024 | worse | 0.4634 | PRDX5 | worse | 0.4164 |
| GPX1_ILMN_1787412 | better | 0.4984 | GPX1 | worse | 0.3078 |
| HBG2_ILMN_2084825 | worse | 0.5201 | HBG2 | better | 0.6453 |
| MB_ILMN_2364864 | worse | 0.5334 | MB | worse | 0.0083 |
| ALB_ILMN_1682763 | worse | 0.5341 | ALB | worse | 0.1134 |
| HP_ILMN_1812433 | worse | 0.5615 | HP | better | 0.6520 |
| TXNRD2_ILMN_1653904 | better | 0.5629 | TXNRD2 | better | 0.0378 |
| GPX1_ILMN_1749662 | better | 0.5751 | GPX1 | worse | 0.3078 |
| PARK7_ILMN_1744713 | better | 0.6230 | PARK7 | better | 0.9375 |
| GPX5_ILMN_1775871 | worse | 0.6398 | GPX5 | NA | NA |
| PRDX5_ILMN_1711606 | worse | 0.6752 | PRDX5 | worse | 0.4164 |
| FABP1_ILMN_1678965 | better | 0.7145 | FABP1 | worse | 0.1431 |
| GPX8_ILMN_3242038 | worse | 0.7218 | GPX8 | worse | 0.0054 |
| GSTZ1_ILMN_1711642 | better | 0.7380 | GSTZ1 | better | 0.3781 |
| PRDX1_ILMN_2366388 | better | 0.7410 | PRDX1 | worse | 0.0162 |
| SOD2_ILMN_1792922 | worse | 0.7940 | SOD2 | worse | 0.0033 |
| GSTZ1_ILMN_1684168 | worse | 0.8022 | GSTZ1 | better | 0.3781 |
| UBIAD1_ILMN_1651872 | worse | 0.8184 | UBIAD1 | better | 0.2936 |
| GPX5_ILMN_2385416 | worse | 0.8273 | GPX5 | NA | NA |
| GSTO1_ILMN_1808196 | worse | 0.8274 | GSTO1 | worse | 0.2851 |
| GSTP1_ILMN_1679809 | worse | 0.8425 | GSTP1 | worse | 0.0205 |
| PRDX6_ILMN_1803180 | worse | 0.8497 | PRDX6 | worse | 0.0410 |
| GPX4_ILMN_1734353 | worse | 0.8861 | GPX4 | better | 0.0271 |
| LTC4S_ILMN_1813335 | worse | 0.8940 | LTC4S | better | 0.0006 |
| SESN2_ILMN_1751598 | worse | 0.9244 | SESN2 | better | 0.0105 |
| MGST1_ILMN_1687387 | better | 0.9319 | MGST1 | worse | 0.0010 |
| MPO_ILMN_1705183 | worse | 0.9375 | MPO | better | 0.9012 |
| DUOX1_ILMN_2292636 | better | 0.9471 | DUOX1 | worse | 0.0160 |
| GPX6_ILMN_1740990 | better | 0.9539 | GPX6 | NA | NA |
| TPO_ILMN_2355423 | better | 0.9631 | TPO | better | 0.5272 |
| MB_ILMN_1766334 | worse | 0.9821 | MB | worse | 0.0083 |
| KDM3B_ILMN_1706539 | better | 0.9834 | KDM3B | worse | 0.2068 |
| MB_ILMN_1748386 | worse | 0.9865 | MB | worse | 0.0083 |
| GSR_ILMN_1775182 | worse | 0.9965 | GSR | worse | 0.2172 |

**Supplementary Table S1.** Association of genes with annotation GO:0016209 ("antioxidant activity") with outcome of pancreatic cancer patients from PACA-AU and TCGA-PAAD. outcome = outcome in cohort with high expression, NA not available
