# Supplementary material for: Mitochondrial superoxide dismutase controls metabolic plasticity in pancreatic cancer
Source: Cell Commun Signal. 2025 Dec 6;23:524. doi: 10.1186/s12964-025-02555-8 (PMC12690781; doi:10.1186/s12964-025-02555-8)
Supplement: Supplementary file 6 — Supplementary Material 6. Supplementary Table S2. Results of gsva analysis of Msigdb hallmark genesets between control KrasG12D and KrasG12D∆Sod2 cell lines. [file 12964_2025_2555_MOESM6_ESM.pdf]

pathway	direction	mean_log2FC	p-value
HALLMARK_MYC_TARGETS_V1	up_in_KrasG12DΔSod2	0.582	0.007
HALLMARK_UNFOLDED_PROTEIN_RESPONSE	up_in_KrasG12DΔSod2	0.293	0.014
HALLMARK_E2F_TARGETS	up_in_KrasG12DΔSod2	0.167	0.037
HALLMARK_ALLOGRAFT_REJECTION	up_in_KrasG12DΔSod2	0.246	0.067
HALLMARK_MYC_TARGETS_V2	up_in_KrasG12DΔSod2	0.527	0.082
HALLMARK_G2M_CHECKPOINT	up_in_KrasG12DΔSod2	0.130	0.273
HALLMARK_MTORC1_SIGNALING	up_in_KrasG12DΔSod2	0.068	0.504
HALLMARK_DNA_REPAIR	up_in_KrasG12DΔSod2	0.088	0.525
HALLMARK_SPERMATOGENESIS	up_in_KrasG12DΔSod2	0.046	0.561
HALLMARK_UV_RESPONSE_UP	up_in_KrasG12DΔSod2	0.040	0.584
HALLMARK_TGF_BETA_SIGNALING	up_in_KrasG12DΔSod2	0.048	0.658
HALLMARK_REACTIVE_OXYGEN_SPECIES_PATHWAY	up_in_KrasG12DΔSod2	0.052	0.817
HALLMARK_COAGULATION	up_in_KrasG12DΔSod2	0.027	0.839
HALLMARK_HEDGEHOG_SIGNALING	down_in_KrasG12DΔSod2	-0.172	0.001
HALLMARK_APICAL_SURFACE	down_in_KrasG12DΔSod2	-0.264	0.004
HALLMARK_GLYCOLYSIS	down_in_KrasG12DΔSod2	-0.171	0.003
HALLMARK_KRAS_SIGNALING_DN	down_in_KrasG12DΔSod2	-0.114	0.027
HALLMARK_HYPOXIA	down_in_KrasG12DΔSod2	-0.184	0.040
HALLMARK_ESTROGEN_RESPONSE_EARLY	down_in_KrasG12DΔSod2	-0.120	0.049
HALLMARK_CHOLESTEROL_HOMEOSTASIS	down_in_KrasG12DΔSod2	-0.405	0.090
HALLMARK_ANGIOGENESIS	down_in_KrasG12DΔSod2	-0.234	0.068
HALLMARK_PROTEIN_SECRETION	down_in_KrasG12DΔSod2	-0.218	0.058
HALLMARK_NOTCH_SIGNALING	down_in_KrasG12DΔSod2	-0.193	0.094
HALLMARK_ANDROGEN_RESPONSE	down_in_KrasG12DΔSod2	-0.173	0.078
HALLMARK_APICAL_JUNCTION	down_in_KrasG12DΔSod2	-0.162	0.098
HALLMARK_HEME_METABOLISM	down_in_KrasG12DΔSod2	-0.151	0.097
HALLMARK_COMPLEMENT	down_in_KrasG12DΔSod2	-0.093	0.116
HALLMARK_FATTY_ACID_METABOLISM	down_in_KrasG12DΔSod2	-0.227	0.129
HALLMARK_BILE_ACID_METABOLISM	down_in_KrasG12DΔSod2	-0.265	0.161
HALLMARK_INTERFERON_GAMMA_RESPONSE	down_in_KrasG12DΔSod2	-0.198	0.157
HALLMARK_IL2_STAT5_SIGNALING	down_in_KrasG12DΔSod2	-0.170	0.150
HALLMARK_TNFA_SIGNALING_VIA_NFKB	down_in_KrasG12DΔSod2	-0.106	0.156
HALLMARK_WNT_BETA_CATENIN_SIGNALING	down_in_KrasG12DΔSod2	-0.194	0.195
HALLMARK_PEROXISOME	down_in_KrasG12DΔSod2	-0.163	0.207
HALLMARK_KRAS_SIGNALING_UP	down_in_KrasG12DΔSod2	-0.116	0.212
HALLMARK_ADIPOGENESIS	down_in_KrasG12DΔSod2	-0.194	0.226
HALLMARK_UV_RESPONSE_DN	down_in_KrasG12DΔSod2	-0.152	0.250
HALLMARK_APOPTOSIS	down_in_KrasG12DΔSod2	-0.141	0.248
HALLMARK_PANCREAS_BETA_CELLS	down_in_KrasG12DΔSod2	-0.255	0.270
HALLMARK_MYOGENESIS	down_in_KrasG12DΔSod2	-0.063	0.291
HALLMARK_INFLAMMATORY_RESPONSE	down_in_KrasG12DΔSod2	-0.058	0.302
HALLMARK_P53_PATHWAY	down_in_KrasG12DΔSod2	-0.042	0.351
HALLMARK_INTERFERON_ALPHA_RESPONSE	down_in_KrasG12DΔSod2	-0.158	0.414
HALLMARK_XENOBIOTIC_METABOLISM	down_in_KrasG12DΔSod2	-0.084	0.422
HALLMARK_IL6_JAK_STAT3_SIGNALING	down_in_KrasG12DΔSod2	-0.066	0.445
HALLMARK_OXIDATIVE_PHOSPHORYLATION	down_in_KrasG12DΔSod2	-0.133	0.548
HALLMARK_PI3K_AKT_MTOR_SIGNALING	down_in_KrasG12DΔSod2	-0.057	0.644
HALLMARK_ESTROGEN_RESPONSE_LATE	down_in_KrasG12DΔSod2	-0.046	0.640
HALLMARK_EPITHELIAL_MESENCHYMAL_TRANSITION	down_in_KrasG12DΔSod2	-0.031	0.741
HALLMARK_MITOTIC_SPINDLE	down_in_KrasG12DΔSod2	-0.032	0.873

Supplementary Table S2. Results of gsva analysis of Msigdb hallmark genesets between control KrasG12D and KrasG12DΔSod2 cell lines.
